# Measuring the well-being of people with dementia: a conceptual scoping review

**DOI:** 10.1186/s12955-020-01440-x

**Published:** 2020-07-24

**Authors:** Chris Clarke, Bob Woods, Esme Moniz-Cook, Gail Mountain, Laila Øksnebjerg, Rabih Chattat, Ana Diaz, Dianne Gove, Myrra Vernooij-Dassen, Emma Wolverson

**Affiliations:** 1 Faculty of Health Sciences, University of Hull, Hull, HU6 7RX UK; 2 DSDC Wales, Bangor University, Ardudwy, Holyhead Road, Bangor, Gwynedd LL57 2PZ UK; 3 School of Dementia Studies, University of Bradford, Richmond Rd, Bradford, BD7 1DP UK; 4 Danish Dementia Research Centre, Rigshospitalet, University of Copenhagen, Section 6922, Blegdamsvej 9, DK-2100 Copenhagen, Denmark; 5 Department of Psychology Università di Bologna - Via Zamboni, 33 - 40126 Bologna, Italy; 6 Alzheimer Europe, L-1417 14, rue Dicks, Luxembourg; 7 Radboud University Medical Centre, Scientific Center for Quality of Healthcare, IQ Healthcare 114, PO Box 9101, 6500HB Nijmegen, The Netherlands

**Keywords:** Dementia, Outcome measurement, Well-being, Quality of life, Positive psychology, Successful aging, Lived experience

## Abstract

**Background:**

Enabling people with dementia to ‘live well’ is a policy and research priority in many countries. However, instruments for measuring outcomes of psychosocial interventions designed to promote well-being in dementia are often derived from a symptom-focused, loss/deficit approach, or from broad quality of life concepts. A pan-European dementia working group called for research on the development of an alternative asset/strengths-based conceptual framework of well-being in dementia. This paper takes forward this recommendation by developing such a framework and using this to map relevant self-report outcome measures.

**Methods:**

Three scoping reviews of published studies were conducted iteratively. First, we examined the literature on lived experiences of well-being and quality of life in people with dementia and then the wider dementia literature for application of well-being constructs. The synthesised findings generated conceptual domains of well-being in people with dementia. Corresponding self-report instruments used in dementia research were scoped, categorised within the conceptual framework and their potential value in measuring outcomes for people with dementia was examined.

**Findings:**

Six conceptual domains for the measurement of well-being and 35 self-report instruments that have been used with people with dementia were identified. Six instruments were developed specifically for people with dementia, five were derived from the gerontological literature and 24 from the well-being literature. Fifteen instruments and one sub-scale have been examined for psychometric properties amongst people with dementia. To date, 20 have been used as outcome measures, with seven measuring change over time. A number of identified instruments utilise traditional retrospective Likert-scaling response formats, limiting their potential for use with some groups of people with dementia.

**Conclusion:**

An assets/strengths-based framework is presented, outlining structural domains for selecting self-report measures of well-being in people with dementia. It provides a foundation for enhancing research into processes and outcomes of psychosocial interventions, including instrument development, more precise matching of intervention aims with outcome measurement, and newer technology-based ‘in-the-moment’ measurement.

## Introduction

Internationally, supporting people to live well with dementia has become the focus of varied public health and research initiatives. Living well with a long-term health condition such as dementia implies the ongoing presence of well-being within a supportive social environment, in spite of health-related adversity. However, psychosocial intervention research in dementia has typically focused on the measurement of cognitive function and/or symptom-reduction (e.g. depression/neuropsychiatric symptoms), even in studies that have also incorporated measures of quality of life (QoL) / health-related quality of life (HRQoL).

Whilst many studies demonstrate that people with dementia can give reliable accounts of their life using existing dementia-specific QoL/HRQoL self-report instruments [1, 2], such measures do not capture the full range of psychosocial outcomes that people with dementia themselves consider important, such as autonomy [3]. Recent studies on how people with dementia might live well with the condition indicate that asset-based factors such as self-efficacy and humour contribute significantly to overall well-being [4, 5]. These factors are closely aligned to the concept of well-being but are not fully captured by traditional Qol/HRQoL instruments.

Moreover, the constructs of QoL and well-being have often been used inter-changeably in dementia research, raising concern about the construct validity of QoL scales [6]. For example, the DEMQOL [7], an established HRQoL measure, subsumes well-being within one of five domains (i.e. ‘health and well-being’), whilst other authors frame ‘subjective well-being’ [8] as related but not equivalent to QoL [9]. Existing research indicates that people with dementia can have a broad range of positive lived experiences [10], influenced by personal and contextual resources [11]. Despite this, a conceptually driven approach to the measurement of these experiences, as potential outcomes of asset-building psychosocial interventions (e.g. those aimed at facilitating agency, participation and social engagement), has so far been lacking.

As such, an important challenge lies in how best to conceptualise well-being and its associated measurement in people with dementia, beyond existing approaches based on QoL. Developing clear conceptual frameworks would in turn underpin valid measurement of well-being in dementia [12]. The application of positive psychology [13] and successful/positive aging gerontological concepts [14] has the potential to meet this challenge. From a positive psychology perspective, Dodge and colleagues [15] define well-being in terms of a state of equilibrium existing between personal resources and life challenges that, when achieved, gives rise to positive emotions and psychological health. This provides a conceptual rationale for subsuming subjective QoL experiences within overarching domains of psychological and social well-being [16, 17], an approach that has synergies with Kitwood’s landmark conceptualisation of well-being and personhood in dementia [18] (see page 8). Additionally, despite the challenges of age-related chronic health conditions, gerontological perspectives show how the successful negotiation of key psychosocial tasks, along with participation and social engagement, can contribute to well-being in terms of successful/positive ageing [19–21].

The application of asset-based perspectives in dementia care is relatively new. A recent trial aimed to improve everyday function through assisting people with dementia to achieve personally meaningful and relevant goals. Significant gains on the primary outcome measure (attainment of these goals) [22] were not mirrored on other outcome measures such as cognition, self-efficacy, mood or dementia-specific HRQoL, demonstrating the insensitivity of current measures to changes personally relevant to people with dementia. Emerging, innovative psychosocial creative and arts-based interventions in dementia that seek to enhance specific aspects of well-being in dementia also require conceptually valid self-report outcome measures to assess accurately their effectiveness [23, 24]. Within this context, a pan-European dementia research programme conducted preliminary work to chart new territory in outcome measurement in dementia [25]. The authors called for further research into asset-based self-report measures. Stoner and colleagues [26] subsequently identified 12 instruments that have been used in research to measure positive psychology constructs in people with dementia. These instruments covered constructs such as identity, hope, optimism, religiosity/spirituality, life valuation, self-efficacy, sense of community and psychological well-being. Such work indicates how, relative to QoL measures, well-being instruments rooted in specific positive constructs offer wider scope and specificity in relation to measuring psychological outcomes in dementia.

The aim of the present study was to extend this work, using scoping reviews to develop an asset/strengths-based conceptual framework for the measurement of well-being in people with dementia, and to use this to map the full range of currently available corresponding self-report instruments that have been used with this population. Our approach is theory-based and empirically-informed, drawing on the successful/positive aging [14] and positive psychology [13] literatures, starting with accounts of lived experiences as the key context for conceptualising well-being in people with dementia.

The specific questions underpinning this review were:

1. What key conceptual domains of well-being can be derived from existing literature involving people with dementia?
2. How have positive psychology and successful / positive aging gerontological concepts been applied to understanding experiences of well-being in people with dementia?
3. What corresponding self-report instruments have been used with people with dementia in published research?

## Methods

We combined principles for scoping reviews [27, 28] with constant comparative methods for analysis [29] to capture the breadth of the literature about well-being in people with dementia, whilst also highlighting current gaps in knowledge. Accordingly, we did not evaluate the methodological quality of studies. We reviewed existing published literature on lived experiences and well-being in people with dementia to synthesise an analytic framework for outcome measurement of well-being. Then we categorised existing self-report instruments used in research involving people with dementia, within corresponding conceptual domains. The PRISMA-ScR checklist [30] for scoping reviews guided these reviews.

**Overall Inclusion/exclusion criteria**: these were based on previous expert consensus work involving several face-to face meetings with one person with dementia, two carers as well as representation from Alzheimer’s Europe (DG & AD) [25]. These criteria were refined by core authors (CC, EM-C, BW & GM) and verified with the wider review team before application. In accordance with the population/concept/context (PCC) framework [31], inclusion criteria were: qualitative and quantitative peer reviewed studies published in the English language, involving strengths, assets and positive experiences of people living with any type or stage of dementia (i.e. population), across all community and clinical settings (i.e. context). To identify relevant instruments, we focused specifically on self-report measures (i.e. context) of different dimensions of well-being, aligned to positive psychology and gerontological / successful ageing constructs (i.e. concept). Exclusion criteria were: grey / non-peer reviewed reports; studies involving non-dementia populations, carers or other dementia care stakeholders (i.e. excluded population) and studies focused or based on a loss/deficit or disability perspective, including studies adopting a symptom-focused approach (i.e. excluded concepts) [31] .

**Study procedures**: study selection involved identifying eligible studies (CC), reviewing their titles/abstracts (CC and EM-C), scrutinising relevant full texts against inclusion criteria, removing duplicates (CC & EM-C), collating and tabulating records (CC) and obtaining independent advice from two authors (BW & GM) in order to resolve ambiguity or uncertainty with regard to study selection and data extraction. Such discussions ensured reliability and occurred at selection and synthesis stages. Independent advice from co-authors (BW & GM) occurred on five occasions; one related to study selection, three in relation to concept synthesis and one regarding instrument mapping.

Three reviews were conducted using the following four steps:

### Step 1: review of reviews - the lived experience of well-being in people with dementia

To obtain an overview of how people with dementia experience aspects of well-being, existing reviews, rather than primary studies, were sought. Web of Science and PsycINFO were searched using the following terms: ‘lived experience’, ‘well-being’, ‘positive experiences’, ‘quality of life’ and ‘dement*’. To be included, reviews had to focus explicitly on lived experiences of well-being and quality of life so that we would be able to develop conceptual domains rooted in the day-to-day experiences of people with dementia. Eligible reviews were therefore scrutinised to exclude associations with the loss/deficit paradigm (e.g. experiences of ‘suffering’ with dementia). Independent arbitration between reviewers was not required at this step. Using thematic synthesis [32], tabulated key findings from each review were used to generate preliminary descriptive themes.

### Step 2: review - application of key well-being concepts in dementia

To refine preliminary themes from step 1 we extended our search to examine our second research question on how positive psychology and successful/positive aging gerontological concepts have been applied to understanding well-being in people with dementia. We systematically searched PsycINFO, MEDLINE and CINAHL-complete for studies relating to concepts of well-being applied to dementia and published before January 2018. The gerontological and positive psychology literature was used to generate the following search terms: dement* combined with *acceptance, autonomy, purpose, self-determination, positive affect, positive emotion, hope, optimism, humor(ur), spirituality, meaning, self-efficacy, self-esteem, self-identity, resilience, belonging, intimacy and social participation* (see Additional File 1). The same inclusion and exclusion criteria as above were applied to capture views and/or experiences of people with dementia expressed via standardised questionnaire measures or qualitative interviews. Studies involving only proxy reports, observational methods or anecdotal case reports were excluded. Studies that had contributed to our first review at Step 1 (i.e. included in selected reviews) were excluded at this step to avoid duplication. Where ambiguity arose, such as inclusion of data from dyads, studies were only included if primary data on the experiences of people with dementia was evident.

Included studies were organised and tabulated. Data extracted from each study were authors, year published, design and methods, number and demographic characteristic of people with dementia, and key findings on well-being in dementia. Self-report instruments used in included studies were also identified and included for consideration at Step 4. Principles of framework analysis [33] were used to categorise findings*,* using the themes derived at Step 1.

### Step 3: synthesis

To develop a conceptual framework of domains relevant to outcome measurement of well-being in dementia, we refined the preliminary themes from Step 1 using narrative synthesis [34] by integrating the findings from qualitative and quantitative studies gathered at Step 2. From this, overarching ‘analytic’ themes [32] were generated using key constructs from the wider well-being literature [8, 15, 16, 35, 36].

### Step 4: review - identifying and categorising self-report measures of well-being in dementia

This third review was to update the inventory of instruments generated from the previous consensus study [25] and align this to proposed conceptual domains of well-being in people with dementia. Studies using self-report measures of well-being in dementia (published before January 2019) were searched for using PsychINFO, MEDLINE, and CINAHL Complete, using terms generated from the theoretical domains developed at Step 3.

The same inclusion and exclusion criteria as above were applied to scrutinise measures of global well-being and/or specific aspects of well-being or successful/positive ageing, including personal strengths and abilities. Hand searching of reference lists and methods sections of dementia studies that had used instruments measuring concepts of well-being, including those taken from studies at Step 2, was also conducted (CC & EM-C) but no other additional sources were used. Studies using a single-item question to measure well-being, rather than a formal instrument, as well as those unavailable in the English Language, [37, 38] were excluded. Instruments based on observation or proxy reports, such as the Music in Dementia Assessment Scales (MiDAS) [39], were also excluded because of the explicit focus on self-reported well-being. All authors reviewed the final list for instruments that may have been missed.

Instruments were categorised according to conceptual domains of well-being in dementia and scrutinised for whether they were developed specifically for people with dementia, and designed as an outcome measure. Studies using each instrument were examined to ascertain if psychometric properties and sensitivity to change had been reported with a dementia sample and whether the instrument had been used in longitudinal or intervention studies. Acceptability and ease of use for people with dementia was also examined, covering aspects such as number of items, response formats, scaling and retrospective judgements.

## Findings

### Lived experiences of well-being in people with dementia

After excluding duplicates and applying limits, searches yielded 200 potentially relevant review articles. Most were excluded because they focused on caregivers or did not review well-being. Of the remaining full text articles, only four systematic reviews directly related to the lived experience of well-being or quality of life in people with dementia. These covered 86 separate studies, totalling 2545 people with dementia (age range 20–100 years; majority female) across community and residential settings, from both developed and developing nations (Additional File 2).

Descriptive themes in lived experiences of well-being in dementia were synthesised into six preliminary themes:

- Feeling Positive: positive emotional states often experienced in the ‘here and now’. Includes hopefulness/humour; positive attitudes - acceptance/ optimism.
- Life Having Meaning: making sense of dementia. Shifting perspectives towards existential meanings such as notions of transcendence/spiritual growth.
- Positive Sense of Self: self-worth, sense of identity (‘I am’) and self-efficacy.
- Keeping Going and Being Active: sense of agency – adaptation/resilience; purpose/ autonomy. Demonstrated by active choices to function ‘normally’ and engage purposefully in meaningful activities which enhance positive emotions.
- Good Relationships: positive aspects of interpersonal and social relationships. Includes attachment and connection (a sense of belonging and safety) as well as feeling valued, loved and accepted by others.
- Feeling Well: a cross-cutting theme of feeling contented and satisfied with life ‘as it is’.

### Application of well-being concepts to dementia

**Fig. 1:**
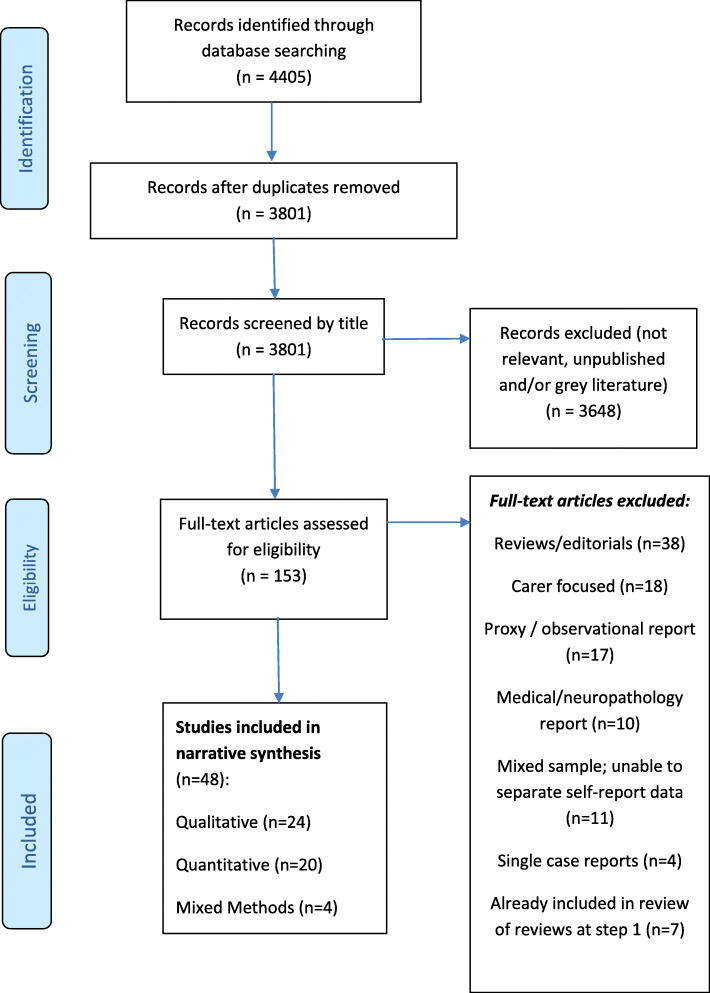
Application of well-being concepts to dementia- PRISMA Diagram for Scoping Review at Step 2

A total of 4405 potentially eligible papers were identified and 153 full-text papers were screened (Fig. 1). The final pool included 48 studies. These originated from several different nations and included a total of 3301 people with dementia (all sub-types). Participants were aged between 54 and 96 years, with the majority over 70 years and female. Settings included: nursing/residential care (*n* = 9); community / out-patients/ memory clinics (*n* = 37) and mixed community / residential care (*n* = 2) (see Additional File 3).

Of the 20 quantitative studies, six involved the evaluation of psychosocial interventions, such as a pilot trial of self-management for people with dementia reporting positive impact on self-reported self-efficacy [40]. Seven were longitudinal, covering topics such as positive affect [41], self-concept [42] and relationship quality [43]. The remaining were cross-sectional designs, investigating specific constructs e.g. spirituality [44], or associations between different concepts such as links between hope, social support and self-esteem [45]. Qualitative studies (*n* = 24) varied in methods and scope, mostly exploring specific constructs, including resilience [46]. Four studies examined the impact of an intervention on general aspects of well-being e.g. perceived benefits of laughter [47] or specific factors such as impact of exercise on self-efficacy [48]. Mixed-methods (*n* = 4) studies covered topics ranging from goal-setting [49] to experiences of friendships [50].

### Synthesis

Five constructs set within the overarching themes of emotional, psychological and social well-being emerged. Examination of studies of hope in people with dementia differentiated hope as a feeling (i.e. positive affect) from the notion of ‘going beyond’ personal goals to find broader meanings and connections, in spite of uncertainty. The sixth broader construct of ‘Valuing Life’ aligned to the construct of life satisfaction [8] and its closer association with quality of life in dementia [9]. Resilience in dementia [46, 51, 52] did not fit easily into one domain. Two studies [46, 51] frame resilience in terms of a strong sense of agency and purpose, achieved through activity and continuity, whilst the third [52] describes experiences of continued social and community engagement as underpinning the construct of resilience. A proposed new framework for the measurement of well-being in dementia can be found in Table 1.

**Table 1:** A conceptual framework for measuring well-being in dementia

Conceptual Theme	Domains
Emotional Well-Being	• **Positive States (*n*** **= 7)**^a^
	Positive affect (e.g. pleasure, enjoyment, contentment); positive experience and associated emotion (e.g. humour) and ‘affect balance’
Psychological Well-Being	• **Going Beyond (n = 7)**^a^
	Personal strengths (e.g. hope) showing aspects of personal growth, meaning-making or spirituality i.e. a sense of transcending the challenges of dementia.
	• **Agency and Purpose (*n*** **= 13)**^a^
	‘Keeping Going’ and remaining’ Active’. Self-determination, autonomy, goals and achievement; ‘resilience’ (defined as remaining strong in the face of dementia or ‘resisting dementia’)’, through continued engagement with meaningful activity.
	• **Positive Sense of Self (n = 13)**^a^
	Positive attitudes toward the self as well as perceived continuation of self-hood, including self-efficacy, self-esteem, sense of identity and dignity.
Social Well-Being	• **Connection and Belonging (n = 6)**^a^
	Experiences of belonging (e.g. close relationships) love, support, appreciation, connection (e.g. meaningful social networks), ‘resilience’ (remaining strong / resisting dementia) through continued social participation, engagement in communities and citizenship.
Life Satisfaction	• **Valuing Life (n = 2)**^a^
	Reflects a general sense of ‘feeling well’ and satisfaction with life as it is e.g. ‘Are you satisfied with your life?’ [53]

^a^ denotes number of studies at Step 2 in each domain

### Self-report instruments for measuring well-being in people with dementia

Thirty-five self-report instruments (of which one instrument has two independently developed sub-scales^1^) used in dementia research were identified and allocated to respective conceptual domains (Table 2). For example, the Gratitude Questionnaire [93] was categorised within ‘Social Well-being: Connection and Belonging’, since gratitude is seen as a social construct [113], whilst the Engagement and Independence in Dementia Questionnaire - EID-Q [77] -, contained just one item for participation in hobbies, and none on social engagement, so this instrument was allocated to ‘Psychological Well-Being: Agency and Purpose’.

**Table 2:** Self -Report Measures of Well-Being used in Existing Dementia Studies

**THEME: EMOTIONAL WELL-BEING**	
• **Positive States n = 3**^a^	
**Dementia Mood Picture Test (DMPT)**^b^ [54]. Measured outcome of a controlled trial of individualised activities within care homes [55].	
**Derogatis Affects Balance Scale (DABS-40)** [56]. Reliability and validity with people with dementia reported in authors’ longitudinal study.	
**Positive and Negative Affect Scale (PANAS-20)** [57]. Measured outcome of a singing intervention in dementia [58].	
**CASP-19: Pleasure Sub-Scale**^c^ [59]. Measured positive quality of life in a population-based cohort study of older people [60]. Validated with a sample of people with dementia [61].	
**THEME: PSYCHOLOGICAL WELL-BEING**	
• **Going Beyond n = 7**^a^	
**Herth Hope Index (HHI-12)** [62]. Measured outcome of spiritual reminiscence intervention in mild-moderate dementia [63] and also feasibility of Dignity Therapy in early stage dementia [64].	
**Life Orientation Test – Revised (LOT-R-10)** [65]. Validated in a cross-sectional early-stage dementia study [66].	
**Meaning in Life Questionnaire** (**MLQ-10**) [67]. Validated in a cross-sectional early stage dementia study [66].	
**Positive Psychology Outcome Measure (PPOM: Hope - 8-item subscale)**^b^;^c^ [68]. Internal consistency and convergent validity with a dementia sample established by authors.	
**Spirituality Index of Well-Being (SIWB-12)** [69]. Measured outcome of spiritual reminiscence intervention in mild-moderate dementia [63].	
**Systems of Belief Inventory (SBI-15)** [70]. Used in an exploratory mixed methods study of spirituality and quality of life in people with dementia [71].	
**Thriving of Older People Assessment Scale (32-item TOPAS)** [72]. Correlates of thriving in dementia explored by authors in a cross-sectional care home study.	
• **Agency and Purpose n = 6**^a^	
**Bangor Goal Setting Interview (BGSI)**^b^ [73]. Measured outcome of goal setting interventions in early stage dementia [22, 49].	
**CASP-19: Control and Autonomy sub-scales.**^c^ [59]. Measures positive quality of life. Used in a population-based cohort study of older people [60]. Psychometric validation study with a dementia sample [61].	
**COOP-WONCA charts of functional status**^c^ [74]. Used in a quality of life study with 67 people, in care homes - some psychometric properties reported [75].	
**Decision Making Involvement scale (DMI-15)**^b^ [76]. Dementia- specific measure of perceived involvement in everyday decision-making - some psychometric properties reported.	
**Engagement and Independence in Dementia Questionnaire (26-item EDI-Q)**^b^ [77]. Dementia specific measure – some psychometric properties reported by scale developers.	
**Positive Psychology Outcome Measure(PPOM: Resilience - 8-item subscale)**^b^; ^c^ [68]. Dementia specific measure - internal consistency /convergent validity established by authors.	
**Resilience Scale (RS-14)** [78]. Part validated in a cross-sectional early stage dementia study [66].	
**Scales of Psychological Well-Being (SPWB):**^c^**Purpose in Life*&*Environmental Mastery sub-scales** [79]. Measured outcome of a retirement home reminiscence intervention [80]. Internal consistency of purpose in life sub-scale reported in a cross-sectional study of goal pursuit in dementia [81].	
• **Positive Sense of Self*n*** **= 6**^a^	
**General Self Efficacy Scale (GSES-10)** [82]. Measured effectiveness in a RCT of self-management in early stage dementia [40].	
**Patient Dignity Inventory (PDI-25)** [83]. Measured outcome of Dignity Therapy in 7 people with dementia [64].	
**Rosenberg Self Esteem Scale (RSES-10**) [84]. Measured outcome of a reminiscence intervention in retirement homes [80] and a multi-modal well-being intervention in early stage dementia [85].	
**Sherer Self Efficacy Scale (SES; 23-items)** [86]. Measured outcome of a choral intervention [58].	
**Self-Identity in Dementia Questionnaire (SID-Q)**^c^ [87]. Originally designed for assessment of role-identity in people with dementia in care homes. Self-report version used in cohort / correlational studies of identity, quality of life, cognition and functional status [88, 89].	
**Scales of Psychological Well-Being (SPWB):**^c^**Self-Acceptance sub-scale** [79]. Measured outcome of a retirement home reminiscence intervention [80].	
**Tennessee Self-Concept Scale (TSCS; 20-item)** [90]. Used in a longitudinal cohort study [42].	
**THEME: SOCIAL WELL-BEING**	
• **Connection and Belonging*n*** **= 7**^a^	
**Brief Sense of Community Scale (BSCS-8)** [91]. Measured outcome of an intergenerational intervention [92].	
**Gratitude Questionnaire (GQ-6)** [93]. Part validated in a cross-sectional early stage dementia study [66].	
**Interpersonal Support Evaluation List (ISEL-12)** [94]. 6-item used to predict hospitalisation in a cohort study [95].	
**Lubben Social Network Scale (LSSN-10)** [96]. Measured outcome of a cooking intervention [97].	
**Mutuality Scale (MS-15)** [98]. Used to predict depression in cohort study [43].	
**Positive Affect Index (5-item PAI)** [99]. Used in a longitudinal study of predictors of relationship-quality over 8 months [100–102]. Preliminary evidence of psychometric properties in dementia [103].	
**Quality of the Current Relationship in Caregiving (QCPR-14)** [104]. Cohort study; stability in scores over 6-month period [105]. Relationship with carer improved for person with dementia in a joint- reminiscence intervention [106]; and an in-home individualised cognitive stimulation therapy [107].	
**Scales of Psychological Well-Being (SPWB):**^c^**Positive relations sub-scale** [79]. Measured outcome of a retirement home reminiscence intervention [80].	
**THEME: LIFE SATISFACTION**	
• **Valuing Life n = 6**^a^	
**CASP-19**^c^ [59]. Four Sub- scales: Control, Autonomy, Self-realisation and Pleasure. 19 and 12-item versions; Used in a population-based cohort study with older people; dementia *n* = 51, but data not separated out [60]. Psychometric validation with a sample of people with dementia [61].	
**Life Satisfaction Index (LSI)** [108]. Adapted 18-item version used as a secondary outcome measure for a spiritual intervention in mild-moderate dementia [63].	
**Satisfaction with Life Scale (5-item SWLS)** [109]. Part validated in a cross-sectional early stage dementia study [66].	
**Short Warwick-Edinburgh Mental Well-Being Scale (7-item SWEMWBS)**^c^ [110]. Includes well-being and positive functioning (over previous 2 weeks). Covers optimism, feeling useful, feeling relaxed**,** dealing with problems, thinking clearly, feeling close to other people and decisiveness.	
**Scales of Psychological Well-Being (SPWB)**^c^ [79]. Six sub-scales: Self –acceptance, positive relations with others, Autonomy, Environmental mastery, Purpose in life, and Personal growth; each with 14-items (other versions available). **‘**Positive relations with Others’ sub-scale used to measure outcome of a retirement home reminiscence intervention [80].	
**WHO-5**^c^ [111]. A 5-item measure of subjective well-being and mood. Adapted to measure a recovery-focussed intervention in early stage dementia [112].	

^a^Denotes number of instruments used in dementia research;^b^ denotes instruments developed specifically for people with dementia; ^c^ denotes instruments that fitted more than one concept and /or domain

Forty studies that have used these instruments with people with dementia were identified. These studies originated from a range of developed and developing nations and collectively included approximately 3676 people with dementia (see Additional File 4. Average reported ages were 72.9 to 91 years. Participants in these studies tended to be female and community dwelling. Nine studies involved people living in residential or institutional settings.

Whilst all included instruments can be construed within an asset/strengths-based perspective on well-being, their conceptual origins varied. For example; the WHO-5 [114] originated from a pragmatic need to measure well-being outcomes in services; the Gratitude Questionnaire GQ-6 [93]; measures a specific positive psychology construct; the Thriving Scale [72] arose from the gerontological literature on well-being; the CASP [59] is presented as a QoL measure but items cover asset-based constructs of well-being. The PANAS [57] specifically measures affect and mood, the PDI dignity instrument [83] arose from the end of life care literature, and the QCPR [104] from caregiving studies.

Only six instruments were specifically developed for people with dementia. These are; the Positive Psychology Outcome Measure - PPOM [68], which comprises separable measures of hope and resilience; the Engagement and Independence in Dementia Questionnaire - EID-Q [77]; the Dementia Mood Picture Test - DMPT [54]; the Decision-Making Involvement Scale - DMI [76]; and the Self-Identity in Dementia Questionnaire - SID-Q [87]. Psychometric properties with dementia populations were reported for only 16 measures but these were variable in scope (see Additional File 4). Particular psychometric weaknesses included sensitivity to change, number of items unsuitable for the full range of people with dementia and complicated response formats.

Nineteen instruments were explicitly developed for outcome measurement. Twenty have been used to measure outcomes in longitudinal (*n* = 4) or intervention (*n* = 16) dementia research, but only three of these have been validated with people with dementia: DMPT [54], DABS [76] and the SPWB - Purpose in Life sub-scale [79] - see Additional File 4. Seven have demonstrated some degree of sensitivity to change in terms of reporting treatment effect. These are: SPWB [79]; WHO-5 [114]; LSI [108] GSE [82]; SIWB-12 [69]; HHI [62]; BGSI [73]. Five have been used in intervention studies although they may not have been designed as outcome measures *per se.* These are: PANAS [57]; RSES-10 [84]; SES-23 [86]; BSCS-8 [91] and the LSI-18 [108]. The full psychometric properties of these instruments for dementia populations have yet to be established.

Seventeen instruments have more than ten items (Additional File 4). Short instruments include the: WHO-5 [114]; GQ-6 [93]; and PAI-5 [99]. Long instruments include the DABS-40 [56] and SPWB-84 [79], although its sub-scales have been used independently [81]. Most instruments have a 4 or 5-point Likert-based response format, but some such as the MLQ-10 [67] extend to 7-Likert points. Four instruments use retrospective response formats, requiring episodic memory, e.g. ‘rate how you have felt over the preceding 2 weeks’. These are the dementia-specific EID-Q and PPOM, [68, 77] the WHO-5, validated for older people [111], and the SWEMWBS [110], which despite its retrospective nature is used as a core outcome measure in many clinical dementia services in the UK [115].

## Discussion

As new theory-driven psychosocial interventions aimed at improving the well-being of the people with dementia emerge, selecting valid instruments to measure their effectiveness remains a challenge. In support, this study establishes the availability of a wide range of self-report instruments that can be used to measure specific aspects of well-being in dementia. In turn, our findings support more precise alignment of outcome measurement to the underlying concepts of a given well-being intervention, as well as aiding research seeking to further understand mechanisms of change.

**Fig. 2:**
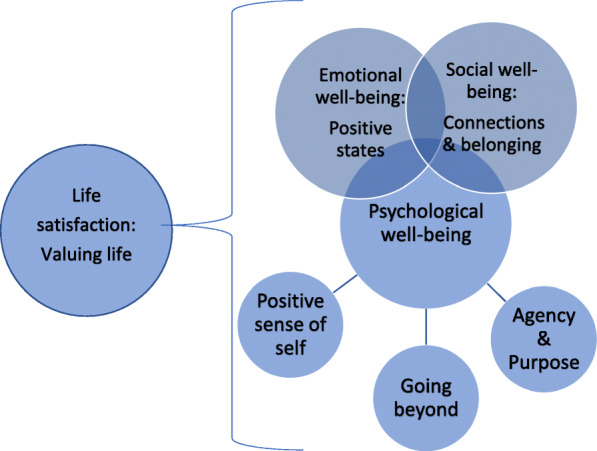
Conceptual map of proposed well-being domains in dementia

This scoping review identified 35 self-report instruments, corresponding to 17 constructs of well-being in dementia, set within 4 overarching domains and 6 sub-domains (see Table 2 & Fig. 2). It builds on a previous systematic review of positive psychology measures (which included quality ratings of instruments), where only 12 instruments used in 17 studies were located [26]. By combining the sucessful/positive aging gerontological literature, we identified several additional instruments (Table 2) that have been used with people with dementia and have potential for instrument development and future research (e.g. WHO-5 well-being index; Spirituality Index of Well-Being; Thriving Scale; Gratitude Scale).

Our findings also expand on a related review [9], which identified only seven measures of well-being taken from six studies, none of which were developed specifically for measuring well-being in people with dementia. The scarcity of studies of well-being and life satisfaction in people with dementia is evident as the authors [9] report insufficient available data for meta-analyses on these two constructs.

Our findings therefore extend the scope of an asset-based conceptualisation of well-being outcome measurement in dementia by highlighting the range and specificity of instruments that could be applied to measuring how people with dementia can be supported to live well via psychosocial interventions and community programmes.

### Psychological, social and emotional well-being

These 3 overlapping and overarching domains for the measurement of well-being in people with dementia (see Fig. 2 – conceptual map of outcomes) resonate with Keyes’ model [35] of well-being. Our framework also concurs with the definition of well-being [15] as a state of equilibrium that, when achieved, gives rise to positive psychological health. Such models propose that positive functioning exists across inter-related domains of well-being, reflecting equilibrium and a potential state of ‘flourishing’ [36], which this review demonstrates can be measurable in spite of the challenges of dementia and multi-morbidities that co-occur in aging.

Evidence for both hedonic (e.g. emotional pleasure; happiness) and eudaimonic [23] well-being in people with dementia can be seen from the synthesised domains. Eudaimonic (psychological) well-being is the most salient overarching domain where, depending on the concept underlying a given intervention, subjective perceptions of positive functioning may be measurable using instruments categorised within the domains of ‘Agency & Purpose’ or ‘Positive Sense of Self’ (Fig. 2).

Of the 16 measures that have been investigated for their psychometric properties with people with dementia, 11 fitted into the overarching domain of psychological well-being, involving agency and associated meaningful engagement. Positive relationships and personal growth across the life span [116] also emerged as aspects of psychological well-being and in studies of lived experiences of well-being in dementia, where some people ‘transcend’ the condition by finding ways to maintain identity and discover new meanings within their experiences [10]. A recent large cohort study found that psychological health (e.g. self-efficacy / positive attitudes towards aging) is strongly associated with subjective perceptions of ‘living well’ in people with dementia [117]. Future research with instruments corresponding to psychological wellbeing could extend emerging knowledge on agency, gratitude, gerotranscendence and personal growth [66, 118–122]. The conceptual domain of psychological well-being and its corresponding instruments (Fig. 2; Table 2) represent an important alternative to existing QoL-based approaches in dementia, which tend not to capture specific and personally meaningful experiences such as hope, self-efficacy and resilience.

Social well-being aligned with the social health-dementia paradigm [123, 124], reflecting ‘Connection and Belonging’ in terms of reciprocity, relationships and social connections. These factors constitute additional potential resources for people with dementia that can help sustain the equilibrium and positive functioning that in turn underpins well-being. Instruments identified in this domain are at an early stage of development; of the 16 measures that have been investigated for their psychometric properties with people with dementia only the 6-item Gratitude Questionnaire [93] relates to social well-being. However, the seven instruments included in this domain provide scope for improving the measurement of positive aspects of reciprocal interpersonal social relationships, participation, citizenship and human rights [125, 126] for people with dementia.

Emotional well-being in dementia has not been well studied, possibly due to biases toward the traditional loss/deficit dominant paradigm of mood disorders. Within this conceptual framework the notion of ‘affect balance’ (i.e. the ratio of positive to negative affect over a specified time period) holds potential for the measurement of emotional well-being in dementia. Kolanowski et al. [127] found that in people with dementia living in nursing homes, those with higher ‘positivity ratios’ had higher levels of social activity, engagement, general well-being and associated resilience. Measurement of affect-balance aligns with the domain of emotional well-being and suggests new territory for the conceptualisation and measurement of resilience in people with dementia.

### Life satisfaction (valuing life)

This fourth overarching conceptual domain of life satisfaction (or ‘Life Valuation’ [26]) emerged as a broader underpinning construct, reflecting overall well-being (Fig. 2), and referring to a general sense of ‘feeling well’ or ‘valuing life’ (Table 2). Studies of well-being and life satisfaction in people with dementia appear to examine different factors apart from mood [9], which may be due to convergence between certain aspects of well-being and emotion [128]. Further research is needed to explore these relationships in the context of living with dementia. The instruments categorised with this domain reflect the complex multi-dimensional nature of well-being. Only two instruments in this domain, the CASP [59, 61] and the SWLS [66, 109] have been validated in dementia to date.

### Resilience

Resilience was embedded across different well-being domains. The construct is represented as agency [51], social connection [52, 129] and emotional resilience [127] in the current literature on living with dementia. Thus resilience, whilst measurable as an outcome in itself [130], can also be conceptualised as a process of ongoing positive adjustment in which people with dementia maintain well-being by utilising various social, psychological and developmental resources as they engage with adversity [131]. Instruments identified for measuring resilience in dementia cover adaptation to the consequences of dementia with agency and purpose [66, 68] but they currently fail to capture the dynamic social processes of continued social and community engagement that are associated with resilience [52, 129].

### Outcome measures for well-being in dementia

None of the instruments developed for people with dementia have yet demonstrated sensitivity to change and none have been formally evaluated for their responsiveness and interpretability i.e. the ability to detect clinically and personally meaningful change in aspects of well-being over time [132].

Self-report outcome measures of well-being should be acceptable and easy to complete for the majority of people with dementia. The measures we identified vary in length (number of items) and complexity of Likert scaling, with some requiring retrospective judgements. This may be problematic for people with difficulties in episodic memory as well as lacking in face validity as people with dementia appear to prefer to report ‘in the moment’ benefits of interventions as they are experienced [133]. This is particularly relevant to capturing meaningful outcomes of interventions such as creative arts-based activities that seek to enhance engagement, social participation and positive affect as they occur [134].

### Limitations

Our review has some limitations. First, despite making every attempt to include the relevant literature, some studies and instruments may not have been identified. This is an inherent problem with iterative scoping review methodology. Second, whilst key information was synthesised and reported in summary tables, statistical techniques were not used to assess methodological quality and we did not formally evaluate the quality of included instruments or examine their psychometric properties. This was in keeping with scoping review methods and the heterogeneity of included studies but also highlights the potential for a future systematic review to address these issues. Third, for some instruments used in dementia studies, it was not possible to categorically define if they had originally been developed as outcome measures *per se*; for example the General Self-Efficacy Scale may be seen as measuring a ‘trait-like’ variable. Examining instrument items is an important consideration when selecting outcome measures intended to be responsive to meaningful changes over time. Fourth, this review only included articles published in English. Fifth, our scope was to chart self-report instruments, so important proxy and observer-rated measures, e.g. MiDAS, [39] were excluded. Finally, we do not provide specific recommendations on measures with strong evidence for use. However, instruments that show validity and potential sensitivity to change in dementia, such as the Herth Hope Index- HHI-12 [62], have good potential for the design of future psychosocial dementia care studies.

## Conclusions

The conceptual framework we present outlines structural domains for selecting self-report measures of well-being in dementia, going beyond QoL/HRQoL instruments used in research to date. It provides a foundation for further research into the process and outcomes of creative well-being enhancing interventions as well as supporting instrument development and the more precise matching of intervention aims with outcome measures. Thirty-five instruments used to date in dementia studies provide a bank of asset-based self-report instruments for future research on well-being in people with dementia. Valid asset/strength-based approaches to the measurement of psychosocial interventions in dementia require good underlying knowledge-frameworks of well-being, which should resonate with the lived experiences of people with dementia.

The next steps in this research agenda include: co-producing [135] new and/or selected key instruments that are matched to meaningful activities and interventions available to people with dementia in their communities; improving and demonstrating the psychometric properties of identified instruments; involving people with dementia in culturally-sensitive outcome measurement [133]; and exploring the potential of technological solutions, such as experience sampling [136], to capture ‘in the moment’ well-being outcomes from interventions. Future research in these areas will elucidate how people with dementia might ‘flourish’ in the ‘here-and-now’, despite the challenges of the condition.

## Supplementary information

**Additional file 1. **Summary Example of Search Strategy (at Step 2; Application of Well-Being Concepts to Dementia). **Data:** Summarised search results.

**Additional file 2.** Review of Reviews - Lived Experiences of Well-Being and Quality of Life in Dementia: Key Findings and Themes. **Data:** Tabulated and synthesised findings.

**Additional file 3 **Domains of well-being in dementia - conceptual synthesis. **Data:** Tabulated and synthesised findings.

**Additional file 4 **. Domain-Based Measures of Wellbeing and Their Use in Dementia (*n* = 35). **Data:** Tabulated and synthesised findings.

## Data Availability

All data generated or analysed during this study are included in this published article [and its supplementary information files].

## Footnotes

1 PPOM sub-scales treated as separate measures as psychometric properties have been established independently for each (see Stoner, 2018a).

## References

[1] Romhild J, Fleischer S, Meyer G, Stephan A, Zwakhalen S, Leino-Kilpi H (2018). Inter-rater agreement of the quality of life-Alzheimer's disease (QoL-AD) self-rating and proxy rating scale: secondary analysis of RightTimePlaceCare data. Health Qual Life Outcomes.

[2] Woods B, Aguirre E, Spector AE, Orrell M (2012). Cognitive stimulation to improve cognitive functioning in people with dementia. Cochrane Database Syst Rev.

[3] Tochel C, Smith M, Baldwin H, Gustavsson A, Ly A, Bexelius C (2019). What outcomes are important to patients with mild cognitive impairment or Alzheimer's disease, their caregivers, and health-care professionals? A systematic review. Alzheimers Dement (Amst).

[4] Lamont RA, Nelis SM, Quinn C, Martyr A, Rippon I, Kopelman MD, Hindle JV, Jones RW, Litherland R, Clare L. Psychological predictors of ‘living well’ with dementia: findings from the IDEAL study. Aging & Mental Health. 2020;24(6):956–64. 10.1080/13607863.2019.1566811 30836765

[5] Reilly ST, Harding AJE, Morbey H, Ahmed F, Williamson PR, Swarbrick C, Leroi I, Davies L, Reeves D, Holland F, Hann M, Keady J. What is important to people with dementia living at home? A set of core outcome items for use in the evaluation of non-pharmacological community-based health and social care interventions. Age Ageing. afaa015. 10.1093/ageing/afaa015.

[6] Bowling A, Rowe G, Adams S, Sands P, Samsi K, Crane M (2015). Quality of life in dementia: a systematically conducted narrative review of dementia-specific measurement scales. Aging Ment Health.

[7] Smith SC, Murray J, Banerjee S, Foley B, Cook JC, Lamping DL (2005). What constitutes health-related quality of life in dementia? Development of a conceptual framework for people with dementia and their carers. Int J Geriatr Psychiatry.

[8] Diener E, Chan MY (2011). Happy people live longer: subjective well-being contributes to health and longevity. Appl Psychol.

[9] Martyr A, Nelis SM, Quinn C, Wu YT, Lamont RA, Henderson C (2018). Living well with dementia: a systematic review and correlational meta-analysis of factors associated with quality of life, well-being and life satisfaction in people with dementia. Psychol Med.

[10] Wolverson EL, Clarke C, Moniz-Cook ED (2016). Living positively with dementia: a systematic review and synthesis of the qualitative literature. Aging Ment Health.

[11] Gorska S, Forsyth K, Maciver D (2018). Living with dementia: a meta-synthesis of qualitative research on the lived experience. The Gerontologist.

[12] Moody CJ, Mitchell D, Kiser G, Aarsland D, Berg D, Brayne C (2017). Maximizing the potential of longitudinal cohorts for research in neurodegenerative diseases: a community perspective. Front Neurosci.

[13] Clarke C, Wolverson E (2016). Positive psychology approaches to dementia.

[14] Cosco TD, Prina AM, Perales J, Stephan BC, Brayne C. Lay perspectives of successful ageing: a systematic review and meta-ethnography. BMJ Open. 2013;3(6):e002710. 10.1136/bmjopen-2013-002710 PMC3686235 23794575

[15] Dodge R, Daly A, Huyton J, Sanders L (2012). The challenge of defining wellbeing. Int J Wellbeing.

[16] Ryff CD, Singer BH (2006). Know thyself and become what you are: a Eudaimonic approach to psychological well-being. J Happiness Stud.

[17] Keyes CLM (1998). Social well-being. Soc Psychol Q.

[18] Kitwood T (1997). Dementia reconsidered: the person comes first. P8.

[19] Pruchno R, Heid AR, Genderson MW (2015). Resilience and successful aging: aligning complementary constructs using a life course approach. Psychol Inq.

[20] Young Y, Frick KD, Phelan EA (2009). Can successful aging and chronic illness coexist in the same individual? A multidimensional concept of successful aging. J Am Med Dir Assoc.

[21] Cornwell B, Laumann EO, Schumm LP (2008). The social connectedness of older adults: a national profile. Am Sociol Rev.

[22] Clare L, Kudlicka A, Oyebode JR, Jones RW, Bayer A, Leroi I (2019). Individual goal-oriented cognitive rehabilitation to improve everyday functioning for people with early-stage dementia: a multicentre randomised controlled trial (the GREAT trial). Int J Geriatr Psychiatry.

[23] Phinney A, Clarke C, Wolverson EL (2016). Well-Being in Dementia. Positive psychology approaches to dementia.

[24] Windle G, Gregory S, Howson-Griffiths T, Newman A, O'Brien D, Goulding A (2018). Exploring the theoretical foundations of visual art programmes for people living with dementia. Dementia.

[25] Mountain G, Moniz-Cook E, Øksnebjerg L. Dementia Outcome Measures: Charting New Territory.2015. Available from: https://www.neurodegenerationresearch.eu/wp-content/uploads/2015/10/JPND-Report-Fountain.pdf1.

[26] Stoner CR, Stansfeld J, Orrell M, Spector A (2019). The development of positive psychology outcome measures and their uses in dementia research: a systematic review. Dementia..

[27] Arksey H, O'Malley L (2005). Scoping studies: towards a methodological framework. Int J Soc Res Methodol.

[28] Levac D, Colquhoun H, O'Brien KK (2010). Scoping studies: advancing the methodology. Implement Sci.

[29] Onwuegbuzie AJ, Leech NL, Collins KM (2012). Qualitative analysis techniques for the review of the literature. Qual Rep.

[30] Tricco AC, Lillie E, Zarin W, O'Brien KK, Colquhoun H, Levac D (2018). PRISMA extension for scoping reviews (PRISMA-ScR): checklist and explanation. Ann Intern Med.

[31] Peters M, Godfrey C, McInerney P, Soares C, Khalil H, Parker D (2015). The Joanna Briggs Institute reviewers' manual 2015: methodology for JBI scoping reviews.

[32] Thomas J, Harden A (2008). Methods for the thematic synthesis of qualitative research in systematic reviews. BMC Med Res Methodol.

[33] Dixon-Woods M (2011). Using framework-based synthesis for conducting reviews of qualitative studies. BMC Med.

[34] Popay J, Roberts H, Sowden A, Petticrew M, Arai L, Rodgers M, Britten N, Roen K, Duffy S (2006). Guidance on the conduct of narrative synthesis in systematic reviews. A product from the ESRC methods Programme Version 1.

[35] Keyes CL (2007). Promoting and protecting mental health as flourishing: a complementary strategy for improving national mental health. Am Psychol.

[36] Seligman MEP (2011). Flourish – a new understanding of happiness and well-being – and how to achieve them.

[37] Muurinen S, Savikko N, Soini H, Suominen M, Pitkala K (2015). Nutrition and psychological well-being among long-term care residents with dementia. J Nutr Health Aging.

[38] Zankd S, Leipold B (2001). The relationship between severity of dementia and subjective well-being. Aging Ment Health.

[39] McDermott O, Orrell M, Ridder HM (2015). The development of music in dementia assessment scales (MiDAS). Nord J Music Ther.

[40] Quinn C, Toms G, Jones C, Brand A, Edwards RT, Sanders F (2016). A pilot randomized controlled trial of a self-management group intervention for people with early-stage dementia (the SMART study). Int Psychogeriatr.

[41] Kolanowski A, Hoffman L, Hofer SM (2007). Concordance of self-report and informant assessment of emotional well-being in nursing home residents with dementia. J Gerontol Ser B Psychol Sci Soc Sci.

[42] Clare L, Whitaker CJ, Nelis SM, Martyr A, Markova IS, Roth I (2013). Self-concept in early stage dementia: profile, course, correlates, predictors and implications for quality of life. Int J Geriatr Psychiatry.

[43] Ball V, Snow AL, Steele AB, Morgan RO, Davila JA, Wilson N (2010). Quality of relationships as a predictor of psychosocial functioning in patients with dementia. J Geriatr Psychiatry Neurol.

[44] Jolley D, Benbow SM, Grizzell M, Willmott S, Bawn S, Kingston P (2010). Spirituality and faith in dementia. Dementia..

[45] Cotter VT, Gonzalez EW, Fisher K, Richards KC (2017). Influence of hope, social support, and self-esteem in early stage dementia. Dementia.

[46] Harris PB. Another wrinkle in the debate about successful aging: The undervalued concept of resilience and the lived experience of dementia. Int J Aging Hum Dev. 2008;67(1):43-61. 10.2190/AG.67.1.c 18630190

[47] Liptak A, Tate J, Flatt J, Oakley MA, Lingler J (2014). Humor and laughter in persons with cognitive impairment and their caregivers. J Holist Nurs.

[48] Olsen CF, Telenius EW, Engedal K, Bergland A (2015). Increased self-efficacy: the experience of high-intensity exercise of nursing home residents with dementia - a qualitative study. BMC Health Serv Res.

[49] Watermeyer TJ, Hindle JV, Roberts J, Lawrence CL, Martyr A, Lloyd-Williams H (2016). Goal setting for cognitive rehabilitation in mild to moderate Parkinson's disease dementia and dementia with Lewy bodies. Parkinsons Dis.

[50] de Medeiros K, Saunders PA, Doyle PJ, Mosby A, Van Haitsma K (2012). Friendships among people with dementia in long-term care. Dementia.

[51] Williamson T, Paslawski T (2016). Resilience in dementia: perspectives of those living with dementia. Can J Speech Lang Pathol Audiol.

[52] Clarke CL, Bailey C (2016). Narrative citizenship, resilience and inclusion with dementia: on the inside or on the outside of physical and social places. Dementia..

[53] Eshkoor S, Hamid TA, SSaH N, Mun CY (2014). Soc Indic Res.

[54] Tappen RM, Barry C (1995). Assessment of affect in advanced Alzheimer's diseases the dementia mood picture test. J Gerontol Nurs.

[55] Kolanowski A, Litaker M, Buettner L, Moeller J, Costa PT (2011). A randomized clinical trial of theory-based activities for the behavioral symptoms of dementia in nursing home residents. J Am Geriatr Soc.

[56] Benedict RHB, Goldstein MZ, Derogatis LR (1996). Assessment of mood states in psychiatrically disturbed patients with dementia. Am J Geriatr Psychiatr.

[57] Watson D, Clark LA, Tellegen A (1988). Development and validation of brief measures of positive and negative affect: the PANAS scales. J Pers Soc Psychol.

[58] Clements-Cortes AA (2013). Buddy's glee Club: singing for life. Act Adapt Aging.

[59] Hyde M, Wiggins RD, Higgs P, Blane DB (2003). A measure of quality of life in early old age: the theory, development and properties of a needs satisfaction model (CASP-19). Aging Ment Health.

[60] Llewellyn DJ, Lang IA, Langa KM, Huppert FA (2008). Cognitive function and psychological well-being: findings from a population-based cohort. Age Ageing.

[61] Stoner CR, Orrell M, Spector A. The psychometric properties of the control, autonomy, self-realisation and pleasure scale (CASP-19) for older adultswith dementia. Aging & Mental Health. 2019;23(5):643–9. 10.1080/13607863.2018.1428940 29356567

[62] Herth K (1992). Abbreviated instrument to measure hope: development and psychometric evaluation. J Adv Nurs.

[63] Wu LF, Koo M (2016). Randomized controlled trial of a six-week spiritual reminiscence intervention on hope, life satisfaction, and spiritual well-being in elderly with mild and moderate dementia. Int J Geriatr Psychiatr.

[64] Johnston B, Lawton S, McCaw C, Law E, Murray J, Gibb J (2016). Living well with dementia: enhancing dignity and quality of life, using a novel intervention, dignity therapy. Int J Older People Nursing.

[65] Scheier MF, Carver CS, Bridges MW (1994). Distinguishing optimism from neuroticism (and trait anxiety, self-mastery, and self-esteem): a reevaluation of the life orientation test. J Pers Soc Psychol.

[66] McGee JS, Zhao HC, Myers DR, Kim SM (2017). Positive psychological assessment and early-stage dementia. Clin Gerontol.

[67] Steger MF, Oishi S, Kashdan TB (2009). Meaning in life across the life span: levels and correlates of meaning in life from emerging adulthood to older adulthood. J Posit Psychol.

[68] Stoner CR, Orrell M, Spector A. The Positive Psychology Outcome Measure (PPOM) for people with dementia: Psychometric properties and factor structure. Arch Gerontol Geriatr. 2018;76:182-7. 10.1016/j.archger.2018.03.001 29529559

[69] Daaleman TP, Frey BB, Wallace D, Studenski SA (2002). Spirituality index of well-being scale: development and testing of a new measure. J Fam Pract.

[70] Holland JC, Kash KM, Passik S, Gronert MK, Sison A, Lederberg M (1998). A brief spiritual beliefs inventory for use in quality of life research in life-threatening illness. Psycho-Oncology..

[71] Katsuno T (2003). Personal spirituality of persons with early-stage dementia: is it related to perceived quality of life?. Dementia.

[72] Bergland A, Kirkevold M, Sandman PO, Hofoss D, Vassbo T, Edvardsson D (2014). Thriving in long-term care facilities: instrument development, correspondence between proxy and residents' self-ratings and internal consistency in the Norwegian version. J Adv Nurs.

[73] Clare L, Nelis S, Kudlicka A (2016). Bangor Goal-Setting Interview Manual.

[74] Van Weel C, Konig-Zahn C, Touw-Otten NP, Van Duijn NP, Meyboom-de JB (1995). Measuring functional health status with the COOP/WONCA charts: a manual. Northern Centre of Health Care Research.

[75] Ettema TP, Hensen E, De Lange J, Droes RM, Mellenbergh GJ, Ribbe MW (2007). Self report on quality of life in dementia with modified COOP/WONCA charts. Aging Ment Health.

[76] Menne HL, Tucke SS, Whitlatch CJ, Feinberg LF (2008). Decision-making involvement scale for individuals with dementia and family caregivers. Am J Alzheimers Dis Other Dement.

[77] Stoner CR, Orrell M, Spector A (2018). Psychometric properties and factor analysis of the engagement and Independence in dementia questionnaire (EID-Q). Dement Geriatr Cogn Disord.

[78] Wagnild GM (2009). The resilience scale user's guide for the US version of the resilience scale and the 14-item resilience scale (RS-14).

[79] Ryff CD, Keyes CLM (1995). The structure of psychological well-being revisited. J Pers Soc Psychol.

[80] Gonzalez J, Mayordomo T, Torres M, Sales A, Melendez JC (2015). Reminiscence and dementia: a therapeutic intervention. Int Psychogeriatr.

[81] Mak W (2010). Self-reported goal pursuit and purpose in life among people with dementia. J Gerontol Ser B Psychol Sci Soc Sci.

[82] Schwarzer R, Jerusalem M (1995). Generalized self-efficacy scale. Weinman J, Wright S, Johnston M, editors.

[83] Chochinov HM, Hassard T, McClement S, Hack T, Kristjanson LJ, Harlos M (2008). The patient dignity inventory: a novel way of measuring dignity-related distress in palliative care. J Pain Symptom Manag.

[84] Rosenberg M (1965). Society and the adolescent self-image.

[85] Burgener SC, Yang Y, Gilbert R, Marsh-Yant S (2008). The effects of a multimodal intervention on outcomes of persons with early-stage dementia. Am J Alzheimers Dis Other Dement.

[86] Sherer M, Maddux JE, Mercandante B, Prentice-Dunn S, Jacobs B, Rogers RW (1982). The self-efficacy scale: construction and validation. Psychol Rep.

[87] Cohen-Mansfield J, Golander H, Arnheim G (2000). Self-identity in older persons suffering from dementia: preliminary results. Soc Sci Med.

[88] Caddell LS, Clare L (2013). How does identity relate to cognition and functional abilities in early-stage dementia?. Neuropsychol Dev Cogn Section B Aging Neuropsychol Cogn.

[89] Caddell LS, Clare L. A profile of identity in early-stage dementia and a comparison with healthy older people. Aging Ment Health. 2013;17(3):319–27. Epub 2012/11/23. 10.1080/13607863.2012.742489 23171274

[90] Fitts WH, Warren WL. Tennessee self-concept scale: TSCS-2 (manual; p.118). Los Angeles: Western Psychological Services; 1996.

[91] Peterson NA, Speer PW, McMillan DW (2008). Validation of a brief sense of community scale: confirmation of the principal theory of sense of community. J Commun Psychol.

[92] Low L-F, Russell F, McDonald T, Kauffman A (2015). Grandfriends, an intergenerational program for nursing-home residents and preschoolers: a randomized trial. J Intergenerational Relationships.

[93] McCullough ME, Emmons RA, Tsang J-A (2002). The grateful disposition: a conceptual and empirical topography. J Pers Soc Psychol.

[94] Cohen S, Mermelstein R, Kamarck T, Hoberman HM, Sarason I, Sarason B (1985). Measuring the functional components of social support. Social Support: Theory, Research and Applications.

[95] Ennis SK, Larson EB, Grothaus L, Helfrich CD, Balch S, Phelan EA (2014). Association of living alone and hospitalization among community-dwelling elders with and without dementia. J Gen Intern Med.

[96] Lubben J (1988). Assessing social networks among elderly populations. Fam Commun Health.

[97] Murai T, Yamaguchi H. Effects of a cooking program based on brain-activating rehabilitation for elderly residents with dementia in a Roken facility: A randomized controlled trial. Progress in Rehabilitation Medicine. 2017;2:20170004. 10.2490/prm.20170004 PMC7365176 32789211

[98] Archbold PG, Stewart BJ, Greenlick MR, Harvath T (1990). Mutuality and preparedness as predictors of caregiver role strain. Res Nurs Health.

[99] Bengtson VL. Positive Affect Index: Subjective solidarity between parents and children. Res Instrum Social Gerontol. 1982:129–33.

[100] Clare L, Nelis SM, Whitaker CJ, Martyr A, Markova IS, Roth I (2012). Marital relationship quality in early-stage dementia: perspectives from people with dementia and their spouses. Alzheimer Dis Assoc Disord.

[101] Nelis SM, Clare L, Martyr A, Markova I, Roth I, Woods RT (2011). Awareness of social and emotional functioning in people with early-stage dementia and implications for carers. Aging Ment Health.

[102] Woods RT, Nelis SM, Martyr A, Roberts J, Whitaker CJ, Markova I (2014). What contributes to a good quality of life in early dementia? Awareness and the QoL-AD: a cross-sectional study. Health Q Life Outcomes.

[103] Woods RT (2009). Relationship quality and quality of life in dementia. 19th International Congress of Gerontology; Paris.

[104] Spruytte N, Van Audenhove C, Lammertyn F, Storms G (2002). The quality of the caregiving relationship in informal care for older adults with dementia and chronic psychiatric patients. Psychol Psychother.

[105] Spector A, Orrell M, Charlesworth G, Marston L (2016). Factors influencing the person-carer relationship in people with anxiety and dementia. Aging Ment Health.

[106] Woods RT, Orrell M, Bruce E, Edwards RT, Hoare Z, Hounsome B (2016). REMCARE: pragmatic multi-Centre randomised trial of reminiscence groups for people with dementia and their family Carers: effectiveness and economic analysis. PLoS One.

[107] Orrell M, Yates L, Leung P, Kang S, Hoare Z, Whitaker C (2017). The impact of individual cognitive stimulation therapy (iCST) on cognition, quality of life, caregiver health, and family relationships in dementia: a randomised controlled trial. PLoS Med.

[108] Neugarten BL, Havighurst RJ, Tobin SS (1961). The measurement of life satisfaction. J Gerontol.

[109] Diener E, Emmons RA, Larsen RJ, Griffin S (1985). The satisfaction with life scale. J Pers Assess.

[110] Stewart-Brown S, Tennant A, Tennant R, Platt S, Parkinson J, Weich S (2009). Internal construct validity of the Warwick-Edinburgh mental well-being scale (WEMWBS): a Rasch analysis using data from the Scottish health education population survey. Health Qual Life Outcomes.

[111] Heun R, Burkart M, Maier W, Bech P (1999). Internal and external validity of the WHO well-being scale in the elderly general population. Acta Psychiatr Scand.

[112] Jha A, Jan F, Gale T, Newman C (2013). Effectiveness of a recovery-orientated psychiatric intervention package on the wellbeing of people with early dementia: a preliminary randomised controlled trial. Int J Geriatr Psychiatr.

[113] McCullough ME, Kimeldorf MB, Cohen AD (2008). An adaptation for altruism. Curr Dir Psychol Sci.

[114] World Health Organization(WHO) (1998). Wellbeing Measures in Primary Health Care: The DepCare Project.

[115] Allward C, Dunn R, Forshaw G, Rewston C, Wass N (2020). Mental wellbeing in people with dementia following cognitive stimulation therapy: innovative practice. Dementia..

[116] Araujo L, Ribeiro O, Paúl C (2017). Hedonic and eudaimonic well-being in old age through positive psychology studies: a scoping review. Anales de Psicología.

[117] Clare L, Wu YT, Jones IR, Victor CR, Nelis SM, Martyr A (2019). A comprehensive model of factors associated with subjective perceptions of "living well" with dementia: findings from the IDEAL study. Alzheimer Dis Assoc Disord.

[118] Bosco A, Schneider J, Coleston-Shields DM, Jawahar K, Higgs P, Orrell M. Agency in dementia care: systematic review and meta-ethnography. Int Psychogeriatr. 2019;31(5):627–42. 10.1017/S1041610218001801 30520403

[119] Pearson MJ (2017). Considering an alternative perspective: an exploration of the meaning and experience of gratitude for individuals living with illness (doctoral dissertation, University of Hull).

[120] Tornstam L (2011). Maturing into gerotranscendence. J Transpersonal Psychol.

[121] Vitale SA, Shaffer CM, Acosta Fenton HR (2014). Self-transcendence in Alzheimer's disease: the application of theory to practice. J Holist Nurs.

[122] Patterson KM (2015). Responses to dementia: a qualitative exploration of self and others (doctoral dissertation, University of Hull).

[123] Vernooij-Dassen M, Jeon YH (2016). Social health and dementia: the power of human capabilities. Int Psychogeriatr.

[124] Vernooij-Dassen M, Moniz-Cook E, Jeon YH (2018). Social health in dementia care: harnessing an applied research agenda. Int Psychogeriatr.

[125] Cahill S (2018). Dementia and human rights.

[126] Dixon J, Laing J, Valentine C. A human rights approach to advocacy for people with dementia: A review of current provision in England and Wales.Dementia. 2020;19(2):221-36. 10.1177/1471301218770478 29665723

[127] Kolanowski AM, Van Haitsma K, Meeks S, Litaker M (2014). Affect balance and relationship with well-being in nursing home residents with dementia. Am J Alzheimers Dis Other Dement.

[128] Berlin M, Fors CF (2019). The association between life satisfaction and affective well-being. J Econ Psychol.

[129] Harris PB. Resilience and Living Well with Dementia. In: Clarke C, Wolverson E, editors. Positive Psychology Approaches to Dementia. London: Jessica Kingsley Publishers; 2016. p. 133-51.

[130] Smith JL, Hanni AA (2019). Effects of a savoring intervention on resilience and well-being of older adults. J Appl Gerontol.

[131] Ryff CD, Singer B (2003). Flourishing under fire: Resilience as a prototype of challenged thriving.

[132] Terwee CB, Bot SD, de Boer MR, van der Windt DA, Knol DL, Dekker J (2007). Quality criteria were proposed for measurement properties of health status questionnaires. J Clin Epidemiol.

[133] Oksnebjerg L, Diaz-Ponce A, Gove D, Moniz-Cook E, Mountain G, Chattat R (2018). Towards capturing meaningful outcomes for people with dementia in psychosocial intervention research: a pan-European consultation. Health Expect.

[134] MacPherson S, Bird M, Anderson K, Davis T, Blair A (2009). An art gallery access programme for people with dementia: 'you do it for the moment'. Aging Ment Health.

[135] Morbey H, Harding AJE, Swarbrick C, Ahmed F, Elvish R, Keady J (2019). Involving people living with dementia in research: an accessible modified Delphi survey for core outcome set development. Trials..

[136] van Knippenberg RJM, de Vugt ME, Ponds RW, Myin-Germeys I, Verhey FRJ (2018). An experience sampling method intervention for dementia caregivers: results of a randomized controlled trial. Am J Geriatr Psychiatr.

