# Supplementary material for: Measuring the well-being of people with dementia: a conceptual scoping review
Source: Health Qual Life Outcomes. 2020 Jul 24;18:249. doi: 10.1186/s12955-020-01440-x (PMC7382062; doi:10.1186/s12955-020-01440-x)
Supplement: Supplementary file 1 — Additional file 1. Summary Example of Search Strategy (at Step 2; Application of Well-Being Concepts to Dementia). Data: Summarised search results. [file 12955_2020_1440_MOESM1_ESM.docx]

**ADDITIONAL FILE 1**

**Summary Example of Search Strategy (at Step 2; Application of Well-Being Concepts to Dementia).**

**Search carried out in PsycINFO. Studies published prior to January 2018.**

**Population:** people living with dementia (any type, at any stage – NOT carers / caregivers)

**Concepts:** well-being and allied concepts drawn from the fields of positive psychology and gerontology / successful ageing

**Contexts:** all community and residential settings; self-reported well-being (via questionnaires or qualitative interviews); lived experiences; naturalistic cohorts; intervention evaluations; survey-based designs.

| **#** | **Searches / Terms** | **Relevant results (initial hits)** |
| --- | --- | --- |
| 1 | dement* OR Alzheimer’s [title, subject heading] |  |
| 2 | people with dement* [title, subject heading] |  |
| 3 | living with dement* [title, subject heading] |  |
| 4 | 1 OR 2 OR 3 NOT “carer” or “caregiver” [title, subject heading] |  |
| 5 | #4 AND “well-being” or “wellbeing” [title and/or abstract] | 9 (420) |
| 6 | #4 AND “acceptance” [title and/or abstract] | 2 (395) |
| 7 | #4 AND “autonomy” [title and/or abstract] | 12 (586) |
| 8 | #4 AND “belonging” [title and/or abstract] | 3 (175) |
| 9 | #4 AND “hope” [title and/or abstract] | 2 (427) |
| 10 | #4 AND “humour” [title and/or abstract] | 5 (56) |
| 11 | #4 AND “intimacy” [title and/or abstract] | 11 (116) |
| 12 | #4 AND “meaning” [title and/or abstract] | 16 (86) |
| 13 | #4 AND “optimis*” [title and/or abstract] | 0 (54) |
| 14 | #4 AND “positive affect” [title and/or abstract] | 9 (100) |
| 15 | #4 AND “purpose” [title and/or abstract] | 2 (16) |
| 16 | #4 AND “resilience” [title and/or abstract] | 2 (76) |
| 17 | #4 AND “self-determination” [title and/or abstract] | 5 (26) |
| 19 | #4 AND “self-efficacy” [title and/or abstract] | 11 (159) |
| 20 | #4 AND “self-identity” [title and/or abstract] | 19 (139) |
| 21 | #4 AND “self-esteem” [title and/or abstract] | 1 (98) |
| 22 | #4 AND “social participation” [title and/or abstract] | 5 (59) |
| 23 | #4 AND “spirituality” [title and/or abstract] | 15 (115) |
