# Supplementary material for: Measuring the well-being of people with dementia: a conceptual scoping review
Source: Health Qual Life Outcomes. 2020 Jul 24;18:249. doi: 10.1186/s12955-020-01440-x (PMC7382062; doi:10.1186/s12955-020-01440-x)
Supplement: Supplementary file 2 — Additional file 2. Review of Reviews - Lived Experiences of Well-Being and Quality of Life in Dementia: Key Findings and Themes. Data: Tabulated and synthesised findings. [file 12955_2020_1440_MOESM2_ESM.docx]

**ADDITIONAL FILE 2**

**Review of Reviews - Lived Experiences of Well-Being and Quality of Life in Dementia: Key Findings and Themes**

| **Review** | **Aims / Methods** | **Findings** | **Key Descriptive Themes** |
| --- | --- | --- | --- |
| Wolverson, Clarke & Moniz-Cook ([1](#_ENREF_1)) | Systematic literature review and synthesis of positive lived experiences in dementia.  Included 27 qualitative studies published between 1992 and 2015 across 6 nations. Twenty studies included PWD living in the community. Three studies drew participants from residential/nursing care settings. All subtypes and severities of dementia were included. Sample sizes ranged from 2-21 (totalling 439 PWD; age range 50-96, 53% female).  Narrative synthesis methods were used to create an interpretive account of living positively with dementia. | Engaging with life in ageing:   - Positive emotion in the here-and-now - Keeping going (perseverance and agency). - Love and support   Engaging with life with dementia:   - Facing it and Fighting it (i.e. accepting and confronting dementia) - Humour - Hope   Identity and Growth:   - Giving thanks (appreciation and gratitude) - Still being me - Growing and transcending | Positive experiences of life now  Attachment / connection  Agency  Positive sense of self  Meaning & Transcendence |
| O’Rourke & Colleagues ([2](#_ENREF_2)) | A systematic review and meta-synthesis of qualitative studies that have investigated factors perceived by people living with dementia to influence their quality of life.  Included 12 papers published between 1974 and 2012, reporting on 11 studies and involving a total of 345 people with dementia. The majority of participants were aged 65+ and female. Countries included UK, USA, Australia, Canada, Japan, The Netherlands & Ireland. Settings included nursing/residential care, day centres and private homes. All types and severities of dementia were considered.  An inductive approach involving taxonomic analysis, constant comparison and importing of concepts was used to produce an integrated synthesis of findings. | Six ‘critical concepts’ were identified:   1. Connectedness – a sense of positive harmony and attachments. 2. Relationships 3. Agency in life today (feeling purposeful) 4. Wellness perspective (feeling ‘well’) 5. Sense of place (feeling positively attached to environment and society) 6. Happiness (positive experiences of joy, contentment, pleasure) and sadness (e.g. grief) – seen as outcomes of key domains of influences on QoL.   **Connectedness** is described as a critical concept that ran through people’s positive experiences in the areas of relationships, agency, wellness and sense of place. | Attachment / connection  Agency  Feeling well  Positive experience of life now |
| Bradshaw, Playford & Riazi ([3](#_ENREF_3)) | Systematic literature review and synthesis of experiences of ‘living well’ in care homes.  At least 14 of 31 identified studies included people with dementia. In total, 1223 participants were included (aged 20-100) and these included PWD from different residential/nursing home settings and with varying types and severities of dementia. Gender split across included studies varied from 40-100% female. Studies were conducted in Australia, Canada, Ireland, Hong Kong, Iceland, The Netherlands, Norway. Taiwan, USA, UK.  Narrative synthesis and meta-ethnographic methods adapted to produce synthesised account of living well in care. | Acceptance and adaptation to living environment – optimism, self-efficacy and resilience.  Connectedness with others – friendships, feeling valued, having a sense of belonging.  Homelike environment – having control and autonomy.  Caring practices – supportive of self-worth, trust and safety. | Positive attitude  Feeling valued  Agency  Positive sense of self |
| Van der Roest & Colleagues ([4](#_ENREF_4)) | Systematic review of literature concerning the subjective needs of people with dementia. Included 34 papers published between 1985 and 2005. Studies only included if reported on the subjective needs of people with dementia living in community or experiences of dementia or what people with dementia perceive as important for their daily quality of life. In total, the review included 538 PWD (ages ranged from 59-89) from across varied settings including community, day centre, residential/nursing care and hospitals. Countries included Canada, Finland, France, Lithuania, New Zealand, Norway, Japan, UK, USA. | Needs related to well-being, quality of life and coping:   - Needing to be accepted and respected - Needing to find strategies to cope with disabilities - Needing to function normally and still do things one used to - Need to come to terms with the situation (acceptance of dementia and support from others) - Needing information about dementia and the care one might need | Feeling valued  Feeling able  Positive Attitude  Making sense |

PWD; People With Dementia

**References**

1. Wolverson EL, Clarke C, Moniz-Cook ED. Living positively with dementia: a systematic review and synthesis of the qualitative literature. Aging & mental health. 2016;20(7):676-99. Epub 2015/06/17.

2. O'Rourke HM, Duggleby W, Fraser KD, Jerke L. Factors that affect quality of life from the perspective of people with dementia: a metasynthesis. Journal of the American Geriatrics Society. 2015;63(1):24-38. Epub 2015/01/20.

3. Bradshaw SA, Playford ED, Riazi A. Living well in care homes: a systematic review of qualitative studies. Age and ageing. 2012;41(4):429-40. Epub 2012/06/09.

4. van der Roest HG, Meiland FJ, Maroccini R, Comijs HC, Jonker C, Droes RM. Subjective needs of people with dementia: a review of the literature. International psychogeriatrics. 2007;19(3):559-92. Epub 2007/01/05.
