# Supplementary material for: Measuring the well-being of people with dementia: a conceptual scoping review
Source: Health Qual Life Outcomes. 2020 Jul 24;18:249. doi: 10.1186/s12955-020-01440-x (PMC7382062; doi:10.1186/s12955-020-01440-x)
Supplement: Supplementary file 3 — Additional file 3 Domains of well-being in dementia - conceptual synthesis. Data: Tabulated and synthesised findings. [file 12955_2020_1440_MOESM3_ESM.docx]

**ADDITIONAL FILE 3**

**Domains of well-being in dementia - conceptual synthesis**

PWD – People With Dementia; CG – Caregiver; AD – Alzheimer’s Disease; LBD - Lewy Bodies Disease; FTD – Fronto-Temporal Dementia; PD – Parkinson’s Disease; PA – Positive Affect; NA – Negative Affect; QoL – Quality of Life; HCs – Healthy Controls; MCI – Mild Cognitive Impairment.

| **Themes from lived experience review of reviews**  **(n=number of relating studies)** | **Key findings from 48 studies that have applied well-being concepts to dementia**   - 20 quantitative (6 intervention/evaluation studies) - 24 qualitative (4 intervention/evaluation studies) - 4 mixed methods (2 intervention/evaluation studies) | | |
| --- | --- | --- | --- |
| ***Feeling Positive***  **(n=7).**  **Synthesised construct:**  ***Positive States*** (positive affect, hope and humour emerging as valued, measurable, and variable over time). | **Details of Study** | **Key Relevant Findings** | **Implications for Thematic Synthesis** |
|  | **Author(s):** Kolanowski et al. [[1](#_ENREF_1)]  **Design/aims:** Quantitative cohort study of concordance between self-and informant-report of positive emotion in dementia.  **Participants:** 31 PWD in nursing care; mean age = 82.7 years; 24 women.  **Context:** Nursing/care homes; USA. | Low levels of concordance between self- and informant ratings of PA. High within-person variability in reports of PA. Stability of PA over time related to cognitive ability and physical functioning. | PA varies over time and experienced idiosyncratically by people with dementia. |
|  | **Author(s):** Cotter et al. [[2](#_ENREF_2)].  **Design/aims:** Quantitative, cross-sectional, correlational study of hope, social support and self-esteem in dementia.  **Participants:** 53 PWD (mean age = 80.7; 68% female).  **Context:** Community; USA. | Self-reported hope predicted self-esteem but unrelated to social support. | Hope in dementia a measurable multi-dimensional state relating to positive experiences of self (also see [[3](#_ENREF_3)]). |
|  | **Author(s):** Kolanowski et al. [[4](#_ENREF_4)]  **Design/aims:** Quantitative, cross-sectional study of positive affect balance and subjective well-being in dementia.  **Participants:** 128 PWD (mean age = 86.1; 77% female).  **Context:** Nursing/care homes; USA. | High levels of overall well-being in approximately 10% of sample. This associated with positive/negative affect ratio of 2.21, i.e. just over 2 instances of positive emotion versus negative within observed time frames. | *Presence* of PA potentially an important component of well-being in dementia. |
|  | **Author(s):** Liptak et al. [[5](#_ENREF_5)].  **Design/aims:** Qualitative focus group study of humour responses to arts engagement activity in dementia.  **Participants:** 20 PWD (aged 60+; 11 women) and primary CGs.  **Context:** Community; USA. | Humour use and humour about dementia prominent throughout interviews - constituted an important coping strategy as well as helping to create a safe social environment. | Humour is a salient positive emotional experience in dementia. |
|  | **Author(s):** Low et al. [[6](#_ENREF_6)].  **Design:** Quantitative; cluster randomised trial of humour therapy in nursing homes.  **Participants:** 310 PWD (mean age = 84.5; 146-161 women, control and interventions groups respectively).  **Context:** Nursing/care homes; Australia. | Engagement in humour therapy associated with increased resident-rated well-being and quality of life. | Humour is present and dynamic for people with dementia. |
|  | **Author(s):** Moos [[7](#_ENREF_7)].  **Design/aims:** Qualitative, linguistic study of humour in conversations in moderate-severe dementia.  **Participants:** 3 PWD (2 women, 1 man; aged 81-89).  **Context:** Nursing/care homes; Scandinavia. | Humour, sarcasm and irony present in verbal communication by PWD, indicating preserved abilities in such areas. | Capacity to experience and express humour may extend into advanced dementia. |
|  | **Author(s):** Wettstein et al. [8]  **Design/aims:** Quantitative, longitudinal study comparing PWD with MCI and HC groups on behavioural functioning and emotional well-being (positive emotion).  **Participants:** Included 35 PWD (mean age = 74.1; 40% female).  **Context:** Community; Israel / Germany. | Lower self-reported PA amongst people with dementia (n=35) relative to a matched, healthy control group - levels of PA stable over 18-month period. | Dementia may jeopardise positive emotion yet stability over time is comparable with people without dementia. |
| **Life Having Meaning**  **(n=7).**  **Synthesised construct:**  ***Going Beyond*** (spiritual experiences and seeking meaning from the creative arts, or personal narratives) | **Author(s):** Beuscher & Grando [[9](#_ENREF_10)].  **Design/aims:** Qualitative study of how spirituality helps people cope with losses in self-esteem, independence, and social interaction in early-stage dementia.  **Participants:** 15 PWD (mean age 78.6; 8 women).  **Context:** Community; USA. | Key themes in using spirituality to cope included; holding onto faith, seeking reassurance and hope, and staying connected. Religious practice and social support enhanced sustained positive attitudes. | Active use of spirituality and religion to cope with perceived and real losses in living with dementia. |
|  | **Author(s):** Byrne & MacKinlay [[1](#_ENREF_11)0].  **Design/aims:** Qualitative study of the impact of meaningful arts-based activities for PWD with depression.  **Participants:** 15 PWD (demographic details not reported).  **Context:** Community; Australia. | Person-centred arts activities have the potential to enhance meaning in life for PWD. Engagement in the activities was a key influence. | Meaning in life linked with creative activities. |
|  | **Author(s):** Kaufman et al. [[1](#_ENREF_12)1].  **Design/aims:** Quantitative - Longitudinal study of impact of spirituality and religion on progression of dementia.  **Participants:** 70 PWD (mean age = 78.4; 69% female).  **Context:** Community – clinic; Canada. | Higher spirituality and religious practices predicted slower progression of cognitive impairment. | Spiritual well-being and religious practice (rather than QoL) impact positively on dementia itself. |
|  | **Author(s):** Jolley [[1](#_ENREF_13)2]  **Design/aims:** Quantitative cross-sectional study. Investigated experiences of spirituality in PWD using a standardised instrument, the Royal Free Interview for Religious and Spiritual Beliefs  **Participants:** 29 PWD (26 women). 50% over 75 years of age.  **Context:** Community / memory clinic; UK. | Spiritual beliefs and religious practices rated as very strong and important in their everyday experiences and coping with dementia | Spirituality and faith are salient well-being factors in dementia. |
|  | **Author(s):** MacKinlay [[1](#_ENREF_14)3].  **Design/aims:** Qualitative analysis of interview and group-work transcripts from Latvian PWD engaged in spiritual reminiscence.  **Participants:** 3 PWD (aged 87-94 years).  **Context:** Nursing home; Latvia. | Key themes included; need for connectedness; spiritual and religious practices; vulnerability and transcendence; physical health issues; wisdom and memory; war experiences; and hope/fear. | Meaning through spirituality is culturally influenced. |
|  | **Author(s):** MacKinlay & Trevitt [[1](#_ENREF_15)4].  **Design/aims:** Examined qualitative data from a mixed methods study of nurse-led spiritual reminiscence in dementia.  **Participants:** 113 PWD (mean age = 83.3; 87% female).  **Context:** Community; Australia. | Two themes studied in relation to the experience and impact of spiritual reminiscence; *meaning in life* and *vulnerability and transcendence.* Group sessions forged closer social connections. | Reminiscence can be spiritually-focused and linked with enhanced meaning and transcendence in dementia. |
|  | **Author(s):** Phinney [[1](#_ENREF_17)5].  **Design/aims:** Qualitative analysis of interviews with 9 PWD, originally focused on perceptions of illness but re-examined to consider meaning and purpose.  **Participants:** 9 PWD (age range 64-88; 5 women).  **Context:** Community; USA. | PWD derived meaning in life from *retained narratives* but, for some, also from *shifting narratives* where they had been able to form new *horizons* of meanings about life since living with dementia. | Meaning in life and link to self-hood might shift in dementia; new meanings can be attained leading to greater self-understanding and well-being. |
| **Keeping Going and Being Active**  **(n=13).**  **Synthesised construct:**  ***Agency and Purpose*** (active engagement in meaningful valued activities; sustaining purpose and a sense of continuity; autonomy and decision- making; agency, choice and resilience). | **Author(s):** Cedervall et al. [[1](#_ENREF_18)6]  **Design/aims:** Qualitative study of personal meanings attached to taking part in physical activity when living with dementia.  **Participants:** 14 PWD (8 women). Age range = 59-79  **Context:** Community; Sweden. | Engagement in physical activity related to maintenance of self-hood. Three key themes in experiences of physical activity; striving to be active; thoughts and beliefs supporting being active; physical activity as a means to well-being. | Ongoing sense of self and well-being as outcomes of engagement in physical activity in dementia. |
|  | **Author(s):** Fetherstonhaugh [[1](#_ENREF_19)7].  **Design/aims:** Qualitative pilot study exploring experience of decision-making in dementia.  **Participants:** 6 PWD (4 women). Age range = 54-78  **Context:** Community; Australia. | Being central to decision making highly valued by participants in terms of their identity and person-hood.  Social support important factor influencing this. | Feeling involved in decision making potentially key to well-being in dementia. |
|  | **Author(s):** Harris [18]  **Design/aims:** Qualitative study to explore experiences of resilience in dementia.  **Participants:** 2 PWD (1 man, aged 71; 1 women, aged 61).  **Context:** Community; USA. | Themes relating to use of assets and protective factors versus risks and vulnerabilities. Participants actively strive to maintain positive assets and sense of purpose and productivity. | Resilience in dementia as an ongoing process and dynamic balance between assets and vulnerabilities. |
|  | **Author(s):** Kaufman & Engel [19].  **Design/aims:** Qualitative study – content analysis used to examine the applicability of a needs-based model of well-being in dementia in the context of residential/nursing care  **Participants:** 19 PWD (mean age = 82.4. 2/3 female).  **Context:** Nursing/care home; Germany | Participants’ perceptions of their well-being reflected key components of Kitwood’s needs-based conceptual model [[2](#_ENREF_22)0] but divergence within each domain was evident. Needs in relation to agency and choice emerged strongly. Subjective health was less of concern in relation to well-being. | Multi-dimensionality of well-being in dementia. Health status one aspect of well-being alongside agency as a key psychological need. |
|  | **Author(s):** Mak [21].  **Design/aims:** Quantitative study exploring relationship between goal pursuit and purpose in life amongst PWD.  **Participants:** 91 PWD (mean age = 75.2; 70 women).  **Context:** Community; USA. | PWD provided reliable self-report data regarding well-being. Strong association between goal pursuit and purpose in life independent of dementia severity. | Sense of purpose achievable through goal-directed activity. |
|  | **Author(s):** Menne & Whitlatch [[2](#_ENREF_23)2].  **Design/aims:** Quantitative, cross-sectional study of predictors of decision-making involvement in PWD  **Participants:** 215 PWD (mean age = 75.9. 50% female).  **Context:** Community, USA. | Predictors of decision-making involvement; younger age, female gender, having more education, having a non-spousal caregiver, fewer months since diagnosis and less impairment in daily activities. Decision making involvement related to less depressive symptoms. | Decision making involvement could vary, reflecting autonomy and well-being. |
|  | **Author(s):** Öhman & Nygård [[2](#_ENREF_24)3].  **Design/aims:** Qualitative study exploring the meanings and motivations behind engagement in daily occupational activities amongst community-dwelling PWD  **Participants:** 6 PWD (3 women, 3 men; age range = 65-80).  **Context:** Community; Sweden. | Engagement in occupations related to opportunities to express autonomy and identity as well as engage in activities without external influence. Occupations connected with efforts to maintain continuity. | Remaining engaged in valued occupations likely to contribute to well-being if autonomy and identity also supported. |
|  | **Author(s):** Phinney [[2](#_ENREF_25)4].  **Design/aims:** Qualitative – multiple interviews with PWD to examine meaning and significance of activity in daily life  **Participants:** 8 PWD (4 women; age range 64-88).  **Context:** Community; Canada. | Being active was a ‘driving force’ in participants’ lives and activities made meaningful via feelings of pleasure and enjoyment; a felt a sense of connection and belonging; and a retained a sense of autonomy and personal identity. | Meaningful activity connected with sense of continuity in positive emotion, belonging and identity. |
|  | **Author(s):** Tak et al. [[2](#_ENREF_26)5].  **Design/aims:** Qualitative study exploring activity engagement in living with dementia.  **Participants:** 37 PWD (mean age = 84.6; 67% female).  **Context:** Community; USA. | Participants remained motivated to engage in self-directed, purposeful and meaningful activities (rather than simply keeping busy) but depended on their social environment to facilitate engagement and experienced barriers to engagement, e.g. fixed schedules. | Self-directed and meaningful activity engagement depend on social environment to support. |
|  | **Author(s):** Stewart-Archer et al. [[2](#_ENREF_27)6]  **Design/aims:** Qualitative study of how PWD define and experience their quality of life.  **Participants:** 136 PWD (mean age = 85.3; 98 women).  **Context:** Community + nursing/care home; Canada. | Having choice/self-determination cut across all domains of subjective quality of life (e.g. health, engaging in activities, independence etc.). | Choice and autonomy are fundamental to how people define their quality of life psychologically. |
|  | **Author(s):** Olsen et al. [[2](#_ENREF_28)7]  **Design/aims:** Qualitative study of experience of exercise in relation to self-efficacy amongst PWD living in nursing care.  **Participants:** 8 PWD (7 women; age range = 69-96).  **Context:** Nursing/care home; Sweden. | A 10-week intensive exercise program enhanced self-efficacy through different mechanisms, including fostering personal investment, empowerment and social connections. | Self-efficacy as an outcome of exercise in dementia. |
|  | **Author(s):** Watermeyer et al. [[2](#_ENREF_29)8].  **Design/aims:** Mixed-methods. Examined feasibility of goal-setting and rating of attainment amongst PWD.  **Participants:** 29 people (6 female) with Lewy Bodies or Parkinson’s dementia (mean age = 75.9).  **Context:** Community / out-patients; UK. | Participants were all able to utilise a structured instrument, the Bangor Goal Setting Interview, see [[29](#_ENREF_30)] to generate personal goals for rehabilitation. | PWD are able to engage with structured instruments for setting goals and are able to use them to rate their perceived attainment of their goals. |
|  | **Author(s):** Williamson & Paslawski [[3](#_ENREF_31)0].  **Design/aims:** Qualitative study examining perspectives on resilience amongst PWD and care-partners.  **Participants:** 7 PWD (+5 care partners). Mean age = 72. Four women, 3 men.  **Context:** Community; Canada. | Three themes in experiences of resilience included active and purposeful living (participation, physical activity and social interaction), perspective (acceptance and openness), and resources (education, support and strategies). These operate together and at different levels in dementia (individual, family, societal). | Resilience relating to ongoing lived experience reflecting interplay between agency and accessing protective psycho-social resources. |
| **Positive Sense of Self**  **(n= 13).**  **Synthesised construct:**  ***Positive Sense of Self***  (retaining identity; maintaining self-esteem, increasing self-efficacy and dignity). | **Author(s):** Batra et al. [[3](#_ENREF_32)1].  **Design/aims:** Qualitative study of narrative self-identity in dementia.  **Participants:** 7 PWD (+7HCs) aged 60-90. PWD; 5 women.  **Context:** Residential care home; USA. | PWD demonstrated self-reference and intact aspects of identity relative to HCs. Time reference was the key difference in identity narratives. | PWD retain aspects of identity and self, separate to memory and awareness impairments. |
|  | **Author(s):** Burgener et al. [[3](#_ENREF_33)2].  **Design/aims:** Quantitative, randomised study of impact of ‘multi-modal’ intervention on self-esteem in dementia.  **Participants:** 43 PWD (mean age = 76-77.9; 20 women).  **Context:** Community; USA. | Evidence of increased self-esteem at 20-weeks attributable to the intervention (control participants’ self-esteem declined). | Increased self-esteem may be an important outcome for psycho-social interventions. |
|  | **Author(s):** Caddell & Clare ([3](#_ENREF_34)3).  **Design/aims:** Quantitative, cross-sectional study exploring sense of identity in  **Participants:** 50 PWD (+age matched HCs); mean age = 77.8; 26 female.  **Context:** Community / memory clinic; UK. | No clear differences between PWD and healthy older people on most aspects of self-rated identity (roles, activities and attributes). | Key aspects of identity are measurable and may not change in early-stage dementia. |
|  | **Author(s):** Clare et al. [[3](#_ENREF_35)4].  **Design/aims:** Quantitative, longitudinal cohort study examining self-concept over time amongst PWD and their care-partners.  **Participants:** 95 PWD (mean age = 78.5; 49 women).  **Context:** Community / memory clinic; UK. | Self-ratings of self-concept were close to average with no significant changes over time. Mood and memory predicted self-concept which predicted quality of life. | Self-concept is usually stable and may be associated with well-being in dementia. |
|  | **Author(s):** Fitzsimmons & Buettner [[3](#_ENREF_36)5].  **Design/aims:** Quantitative evaluation of an educational health-promotion course for PWD  **Participants:** 10 PWD (5 women; mean age = 77.9).  **Context:** Community; USA. | Increase in mean self-esteem scores after the intervention. | Engaging in education and learning may increase self-esteem as an outcome in dementia. |
|  | **Author(s):** Frazier et al. [[3](#_ENREF_37)6].  **Design/aims:** Quantitative cross-sectional study exploring differences in self-representations between PWD (Alzheimer’s and Parkinson’s) and healthy older people.  **Participants:** 34 PWD (mean age = 70.7; 47% female).  **Context:** Community; USA. | Self-efficacy and future outcome expectancies lower both for PWD and PD relative to healthy controls. | Dementia threatens self-efficacy relative to healthy ageing. |
|  | **Author(s):** Johnston et al. [[3](#_ENREF_38)7].  **Design/aims:** Mixed methods – assessed feasibility, acceptability and effectiveness of modified dignity therapy in dementia.  **Participants:** 7 PWD (2 women; age range = 71-82).  **Context:** Community; UK. | Some improvements noted for 4 participants on levels of dignity and hope. Intervention reported to be acceptable and feasible. Key themes in participants’ experiences post-therapy; a life in (historical) context, a ‘key to connect’ (using dignity document), and personal legacy (uncertain future). | Dignity in dementia is measurable and can be enhanced. |
|  | **Author(s):** Marshall et al. [[3](#_ENREF_39)8].  **Design/aims:** Quantitative, pilot RCT of a ‘living well’ programme for PWD. Self-esteem a secondary outcome  **Participants:** 58 PWD (mean age = 74.6-76.6. 33 women).  **Context:** Community / memory clinic; UK. | Evidence of improvement in self-esteem attributable to the group intervention. | Self-esteem in dementia can be improved through structured interventions. |
|  | **Author(s):** Quinn et al. [39].  **Design/aims:** Quantitative – pilot RCT of a self-management group intervention for PWD.  **Participants:** 24 PWD (mean age = 75.2-76; 16 women).  **Context:** Community / memory clinic; UK. | Gains in self-efficacy attributable to the intervention evidenced at 3- and 6-month follow up. | Self-efficacy in dementia as an outcome of enhanced self-management. |
|  | **Author(s):** Sprange et al. [[4](#_ENREF_41)0].  **Design/aims:** Quantitative study – tested feasibility and effectiveness of a community-based self-management for PWD  **Participants:** 10 PWD (5 women; ages ranged from 60s to 80s).  **Context:** Community / memory clinic; UK. | 12-week structured intervention delivered in groups and individually resulted in improved self-efficacy as well as re-engagement in activities and relationships. | Improved self-efficacy a key outcome of self-management interventions in dementia. |
|  | **Author(s):** Tranvag et al. [[4](#_ENREF_42)1].  **Design/aims:** Qualitative – exploration of experiences of dignity interactions in dementia.  **Participants:** 11 PWD (5 women; age range = 64-85).  **Context:** Community / memory clinic; Norway. | Three qualities of interactions maintain personal dignity; experiences of love & confirmation, social inclusion / fellowship, and being treated with warmth and understanding yet still an equal. | Personal experience of dignity relates to quality of social interactions, combining inclusion with support for autonomy. |
|  | **Author(s):** Tranvag et al. [[4](#_ENREF_43)2].  **Design/aims:** Qualitative – exploratory study of the dignity experiences amongst people with dementia and MCI.  **Participants:** 11 PWD (5 women; age range = 64-85).  **Context:** Community / memory clinic; Norway. | Dignity experiences of participants found to have 3 crucial dimensions; historical (including a sense of gratitude to parents/family), intra-personal (recognising one’s personal worth – living according to internal values’) and interpersonal (‘experiencing being part of a caring and confirming communion’). | Dignity as a marker for subjective well-being – psychological and social components. |
|  | **Author(s):** van Gennip et al. [43].  **Design/aims:** Qualitative study – explored how dementia might affect the experience of dignity.  **Participants:** 14 PWD (6 women; age range = 50-94).  **Context:** Community / residential care; The Netherlands. | People experienced a continued sense of dignity but this was diminished because of the impact of cognitive impairment on autonomy and identity, moderated by experiences of supportive social contexts. | Sense of dignity incorporating identity and perceived autonomy of the person. Dignity impacted by social environment. |
| **Good Relationships**  **(n=6)**  **Synthesised construct: *Connection and Belonging***  (personal and intergenerational social identity, where key aspects were social activities, friendships, reciprocity, a sense of belonging to people and places and citizenship). | **Author(s):** Ball et al. [[4](#_ENREF_44)4].  **Design/aims:** Quantitative, prospective cohort study. Investigated relationship between mutuality (relationship quality) and psychological functioning in dementia.  **Participants:** 171 PWD (mean age = 76; 3% female).  **Context:** Community; USA. | Mutuality scores predicted lowered depression and increased positive events over time for PWD. Increased mutuality associated with increased social stimulation over time. | Reciprocity as a way to frame relationship quality in dementia – measurable through self-report. |
|  | **Author(s):** Clare et al. [[4](#_ENREF_45)5].  **Design/aims:** Qualitative study of the impact of shared social identity amongst members of DASNI (Dementia Advocacy and Support International).  **Participants:** 7 PWD (5 women; mean age = 60.2).  **Context:**  Active members of international self-advocacy organisation. | Four key themes; Dementia Land (representing the challenges of dementia), Collective Strength (a sense of belonging and reciprocal support), A Valuable Contributing Member of Society (purpose), and There Is Life After A Diagnosis of Dementia (control). | A shared social identity could enhance well-being – belonging and reciprocal support key aspects. |
|  | **Author(s):** Clarke & Bailey [[4](#_ENREF_46)6].  **Design/aims:** Qualitative, longitudinal study exploring how place and context influence how people narrate their everyday lives and sense of resilience.  **Participants:** 13 PWD (7 women; ages ranged from 60s to 90s).  **Context:** Community; UK. | Narrative citizenship occurred when people were able to retain familiarity and a sense of inclusion with people and places and ‘feel on the inside’. Being able to narrate a story of inclusion and citizenship in turn connected with a sense of resilience and understanding of dementia. Key (positive) themes included; others knowing and responding, sustaining and changing activities, belonging, engaging supports. | Resilience in dementia supported by experiences of inclusive social participation, belonging and citizenship. |
|  | **Author(s):** de Madeiros et al. [[4](#_ENREF_47)7].  **Design/aims:** Mixed-methods longitudinal study of friendships involving 31 PWD in supported living environment.  **Participants:** 31 PWD (mean age = 82.4; 21 women).  **Context:** Residential care; USA. | Disparities emerged between how PWD identified and described friendships and how this was done by care staff. PWD able to identify and discuss friendships and their value. | The subjective experience of friendships in dementia – sustained salience. |
|  | **Author(s):** Han & Radel [[4](#_ENREF_48)8]  **Design/aims:** Qualitative study of the impact and experience of an intergenerational social activities programme for people with dementia.  **Participants:** 5 PWD (2 women; age range = 61-89).  **Context:** Community; USA. | Three themes identified through Interpretive Phenomenological Analysis (IPA) covered enjoyment of social activities that support self-identity, valuing new relationships and friendships, and empowerment (gaining choice and control). | Social relationships and activities across generations bringing positive emotions. |
|  | **Author(s):** Phinney et al. [[50](#_ENREF_49)].  **Design/aims:** Qualitative, ethnographic study of experiences of social citizenship through a community-based activity support group  **Participants:** 12-15 PWD; members of a community walking group. Demographic details not reported.  **Context:** Community (walking group); Canada. | Participants co-construct a sense of social citizenship through the shared experience of regular walking in their neighbourhood. Three themes in experiences were; keeping the focus off dementia, creating a place of belonging, and claiming a place in the community. | Shared social activities foster connection with communities - improved social aspects of subjective well-being could be captured as outcomes. |
| **Feeling well**  **(n=2)**  **Synthesised construct: *Life Satisfaction - Valuing Life.*** | **Author(s):** Eshkoor et al. [[50](#_ENREF_50)].  **Design/aims:** Quantitative, cross-sectional study of predictors of life satisfaction amongst older people with cognitive impairment in Malaysia.  **Participants:** 1210 PWD (aged 60+ years; gender unreported).  **Context:** Community; Malaysia. | 83% of sample reported being satisfied with their lives. Social support a key predictor of expressed life satisfaction. | Life satisfaction as a measurable aspect of well-being in dementia - influenced by social relationships and support. |
|  | **Author(s):** Zankd & Leipold [[5](#_ENREF_51)1].  **Design/aims:** Quantitative, cross sectional study of relationships between cognitive ability, health, social support, depression and life satisfaction in dementia.  **Participants:** 63 PWD (mean age = 79.2; 76% female).  **Context:** Community (day care centres); Germany. | Physical health moderates relationship between cognitive status and LS. Depression and LS inversely related. | Self-reported life satisfaction as a measurable outcome and related to physical and psychological health.  N.B. Life Satisfaction Questionnaire [[5](#_ENREF_52)2] is unavailable in English. |

**References**

1. Kolanowski A, Hoffman L, Hofer SM. Concordance of self-report and informant assessment of emotional well-being in nursing home residents with dementia. The journals of gerontology Series B, Psychological sciences and social sciences. 2007;62(1):P20-7. Epub 2007/02/08.

2. Cotter VT, Gonzalez EW, Fisher K, Richards KC. Influence of hope, social support, and self-esteem in early stage dementia. Dementia. 2017;17(2):214-24. Epub 2017/11/23.

3. Wolverson EL, Clarke C, Moniz-Cook E. Remaining hopeful in early-stage dementia: a qualitative study. Aging & mental health. 2010;14(4):450-60. Epub 2010/05/11.

4. Kolanowski AM, Van Haitsma K, Meeks S, Litaker M. Affect Balance and Relationship With Well-Being in Nursing Home Residents With Dementia. American journal of Alzheimer's disease and other dementias. 2014;29(5):457-62. Epub 2014/01/11.

5. Liptak A, Tate J, Flatt J, Oakley MA, Lingler J. Humor and laughter in persons with cognitive impairment and their caregivers. Journal of holistic nursing : official journal of the American Holistic Nurses' Association. 2014;32(1):25-34. Epub 2013/08/09.

6. Low LF, Brodaty H, Goodenough B, Spitzer P, Bell JP, Fleming R, et al. The Sydney Multisite Intervention of LaughterBosses and ElderClowns (SMILE) study: cluster randomised trial of humour therapy in nursing homes. BMJ open. 2013;3(1). Epub 2013/01/15.

7. Moos I. Humour, irony and sarcasm in severe Alzheimer's dementia – a corrective to retrogenesis? Ageing and Society. 2011;31(02):328-46.

8. Wettstein M, Seidl U, Wahl H-W, Shoval N, Heinik J. Behavioral Competence and Emotional Well-Being of Older Adults with Mild Cognitive Impairment. GeroPsych. 2014;27(2):55-65.

9. Beuscher L, Grando VT. Using spirituality to cope with early-stage Alzheimer's disease. Western journal of nursing research. 2009;31(5):583-98. Epub 2009/03/14.

10. Byrne L, MacKinlay E. Seeking Meaning: Making Art and the Experience of Spirituality in Dementia Care. Journal of Religion, Spirituality & Aging. 2012;24(1-2):105-19.

11. Kaufman Y, Anaki D, Binns M, Freedman M. Cognitive decline in Alzheimer disease: Impact of spirituality, religiosity, and QOL. Neurology. 2007;68(18):1509-14. Epub 2007/05/02.

12. Jolley D, Benbow SM, Grizzell M, Willmott S, Bawn S, Kingston P. Spirituality and faith in dementia. Dementia. 2010;9(3):311-25.

13. MacKinlay E. Using Spiritual Reminiscence with a Small Group of Latvian Residents with Dementia in a Nursing Home: A Multifaith and Multicultural Perspective. Journal of Religion, Spirituality & Aging. 2009;21(4):318-29.

14. Mackinlay E, Trevitt C. Living in aged care: using spiritual reminiscence to enhance meaning in life for those with dementia. International journal of mental health nursing. 2010;19(6):394-401. Epub 2010/11/09.

15. Phinney A. Horizons of Meaning in Dementia: Retained and Shifting Narratives. Journal of Religion, Spirituality & Aging. 2011;23(3):254-68.

16. Cedervall Y, Torres S, Aberg AC. Maintaining well-being and selfhood through physical activity: experiences of people with mild Alzheimer's disease. Aging & mental health. 2015;19(8):679-88. Epub 2014/10/01.

17. Fetherstonhaugh D, Tarzia L, Nay R. Being central to decision making means I am still here!: the essence of decision making for people with dementia. Journal of aging studies. 2013;27(2):143-50. Epub 2013/04/09.

18. Harris PB. Another wrinkle in the debate about successful aging: the undervalued concept of resilience and the lived experience of dementia. International journal of aging & human development. 2008;67(1):43-61. Epub 2008/07/17.

19. Kaufmann EG, Engel SA. Dementia and well-being: A conceptual framework based on Tom Kitwood's model of needs. Dementia. 2016;15(4):774-88. Epub 2014/06/21.

20. Kitwood T. Dementia reconsidered: The person comes first. Buckingham: Open University Press.; 1997.

21. Mak W. Self-reported goal pursuit and purpose in life among people with dementia. Journals of Gerontology Series B: Psychological Sciences and Social Sciences. 2011 Mar 1;66(2):177-84.

22. Menne HL, Whitlatch CJ. Decision-Making Involvement of Individuals With Dementia. The Gerontologist. 2007;47(6):810-9.

23. Öhman A, Nygård L. Meanings and Motives for Engagement in Self-Chosen Daily Life Occupations among Individuals with Alzheimer's Disease. OTJR: Occupation, Participation and Health. 2005;25(3):89-97.

24. Phinney A, Chaudhury H, O'Connor DL. Doing as much as I can do: the meaning of activity for people with dementia. Aging & mental health. 2007;11(4):384-93. Epub 2007/07/07.

25. Tak SH, Kedia S, Tongumpun TM, Hong SH. Activity Engagement: Perspectives from Nursing Home Residents with Dementia. Educational gerontology. 2015;41(3):182-92. Epub 2014/12/10.

26. Stewart-Archer LA, Afghani A, Toye CM, Gomez FA. Subjective quality of life of those 65 years and older experiencing dementia. Dementia. 2016;15(6):1716-36. Epub 2016/10/30.

27. Olsen CF, Telenius EW, Engedal K, Bergland A. Increased self-efficacy: the experience of high-intensity exercise of nursing home residents with dementia - a qualitative study. BMC health services research. 2015;15:379. Epub 2015/09/16.

28. Watermeyer TJ, Hindle JV, Roberts J, Lawrence CL, Martyr A, Lloyd-Williams H, et al. Goal Setting for Cognitive Rehabilitation in Mild to Moderate Parkinson's Disease Dementia and Dementia with Lewy Bodies. Parkinson's disease. 2016;2016:8285041. Epub 2016/07/23.

29. Clare L, Nelis S, Kudlicka A. Bangor Goal-Setting Interview Manual2016 12/12/2017. Available from: https://psychology.exeter.ac.uk/media/universityofexeter/schoolofpsychology/reach/documents/The_Bangor_Goal-Setting_Interview_Version_2_Manual_(BGSI_v.2)_Dec_2016.pdf

30. Williamson T, Paslawski T. Resilience in dementia: Perspectives of those living with dementia. Canadian Journal of Speech-Language Pathology and Audiology. 2016;40(1):1-15.

31. Batra S, Sullivan J, Williams BR, Geldmacher DS. Qualitative assessment of self-identity in people with advanced dementia. Dementia. 2016;15(5):1260-78. Epub 2015/10/24.

32. Burgener SC, Yang Y, Gilbert R, Marsh-Yant S. The effects of a multimodal intervention on outcomes of persons with early-stage dementia. American journal of Alzheimer's disease and other dementias. 2008;23(4):382-94. Epub 2008/05/06.

33. Caddell LS, Clare L. A profile of identity in early-stage dementia and a comparison with healthy older people. Aging & mental health. 2013;17(3):319-27. Epub 2012/11/23.

34. Clare L, Whitaker CJ, Nelis SM, Martyr A, Markova IS, Roth I, et al. Self-concept in early stage dementia: profile, course, correlates, predictors and implications for quality of life. International journal of geriatric psychiatry. 2013;28(5):494-503.

35. Fitzsimmons S, Buettner LL. Health promotion for the mind, body, and spirit: a college course for older adults with dementia. American journal of Alzheimer's disease and other dementias. 2003;18(5):282-90. Epub 2003/10/23.

36. Frazier LD, Cotrell V, Hooker K. Possible selves and illness: A comparison of individuals with Parkinson's disease, early-stage Alzheimer's disease, and healthy older adults. International Journal of Behavioral Development. 2003;27(1):1-11.

37. Johnston B, Lawton S, McCaw C, Law E, Murray J, Gibb J, et al. Living well with dementia: enhancing dignity and quality of life, using a novel intervention, Dignity Therapy. Int J Older People Nurs. 2016;11(2):107-20. Epub 2015/12/30.

38. Marshall A, Spreadbury J, Cheston R, Coleman P, Ballinger C, Mullee M, et al. A pilot randomised controlled trial to compare changes in quality of life for participants with early diagnosis dementia who attend a 'Living Well with Dementia' group compared to waiting-list control. Aging & mental health. 2015;19(6):526-35. Epub 2014/09/10.

39. Quinn C, Toms G, Jones C, Brand A, Edwards RT, Sanders F, et al. A pilot randomized controlled trial of a self-management group intervention for people with early-stage dementia (The SMART study). International psychogeriatrics. 2016;28(5):787-800. Epub 2015/12/18.

40. Sprange K, Mountain GA, Shortland K, Craig C, Blackburn D, Bowie P, et al. Journeying through Dementia, a community-based self-management intervention for people aged 65 years and over: a feasibility study to inform a future trial. Pilot and feasibility studies. 2015;1:42. Epub 2016/12/15.

41. Tranvag O, Petersen KA, Naden D. Relational interactions preserving dignity experience: Perceptions of persons living with dementia. Nursing ethics. 2015;22(5):577-93. Epub 2014/10/17.

42. Tranvag O, Petersen KA, Naden D. Crucial dimensions constituting dignity experience in persons living with dementia. Dementia. 2016;15(4):578-95. Epub 2014/04/20.

43. van Gennip IE, W. Pasman HR, Oosterveld-Vlug MG, Willems DL, Onwuteaka-Philipsen BD. How dementia affects personal dignity: a qualitative study on the perspective of individuals with mild to moderate dementia. Journals of Gerontology Series B: Psychological Sciences and Social Sciences. 2016 May 1;71(3):491-501.

44. Ball V, Snow AL, Steele AB, Morgan RO, Davila JA, Wilson N, et al. Quality of relationships as a predictor of psychosocial functioning in patients with dementia. Journal of geriatric psychiatry and neurology. 2010;23(2):109-14. Epub 2010/03/18.

45. Clare L, Rowlands JM, Quin R. Collective strength: The impact of developing a shared social identity in early-stage dementia. Dementia. 2008;7(1):9-30.

46. Clarke CL, Bailey C. Narrative citizenship, resilience and inclusion with dementia: On the inside or on the outside of physical and social places. Dementia. 2016;15(3):434-52. Epub 2016/05/14.

47. de Medeiros K, Saunders PA, Doyle PJ, Mosby A, Van Haitsma K. Friendships among people with dementia in long-term care. Dementia. 2012;11(3):363-81.

48. Han A, Radel J. The Benefits of a Person-Centered Social Program for Community-Dwelling People with Dementia: Interpretative Phenomenological Analysis. Activities, Adaptation & Aging. 2017;41(1):47-71.

49. Phinney A, Kelson E, Baumbusch J, O'Connor D, Purves B. Walking in the neighbourhood: Performing social citizenship in dementia. Dementia. 2016;15(3):381-94. Epub 2016/05/14.

50. Eshkoor S, Hamid TA, Nudin SSaH, Mun CY. The Effects of Social Support, Substance Abuse and Health Care Supports on Life Satisfaction in Dementia. Social Indicators Research. 2014;116(2):535-44.

51. Zankd S, Leipold B. The relationship between severity of dementia and subjective well-being. Aging & mental health. 2001;5(2):191-6. Epub 2001/08/21.

52. Closs C, Kempe P. Eine differenzierende Betrachtung und Validierung des Konstruktes Lebenszufriedenheit: Analyse bewährter Verfahren und Vorschläge für ein methodisch fundiertes Vorgehen bei der Messung der Dimension dieses Konstruktes. Zeitschrift für Gerontologi. 1986;19(1):47-55.
