# Supplementary material for: Measuring the well-being of people with dementia: a conceptual scoping review
Source: Health Qual Life Outcomes. 2020 Jul 24;18:249. doi: 10.1186/s12955-020-01440-x (PMC7382062; doi:10.1186/s12955-020-01440-x)
Supplement: Supplementary file 4 — Additional file 4 . Domain-Based Measures of Wellbeing and Their Use in Dementia (n = 35). Data: Tabulated and synthesised findings. [file 12955_2020_1440_MOESM4_ESM.docx]

ADDITIONAL FILE 4: Domain-Based Measures of Wellbeing and Their Use in Dementia (n=35**^[[1]](#footnote-1)^**)

*PWD - People with Dementia; RCT – Randomised Controlled Trial;* α – Cronbach’s alpha

| **Wellbeing domain** | **Instrument** | **Characteristics of Instrument** | **Use with People with Dementia**  [Key illustrative examples; n=40 studies]  **Key:**  * Instrument validated with people living with dementia  Ω Instrument used as an outcome measure in dementia  ^ Instrument showed sensitivity to change in dementia |
| --- | --- | --- | --- |
| **Positive States** | Dementia Mood Picture Test; DPMT [[1](#_ENREF_1)] | A measure of current self-reported positive and negative moods using pictures of facial expressions as stimuli. Developed specifically for people with dementia.  **Response format:** Current - each mood is scored 0–2 by a rater depending on the person’s responses to standardised questions.  **Designed as an outcome measure (Y/N)?** Y | *  Ω  DMPT used as outcome measure in RCT of individualised activities amongst 128 PWD (mean age = 86; 76% female) in residential / nursing environments in the USA - instrument not detect change in response to intervention but achieved high levels of inter-rater reliability (r=.99) [2].  Also used to measure benefits of a memory centre day programme amongst 36 PWD (mean age 80.3; n=17 women) [3]. No mood changes were detected. Cronbach alpha of 0.76 reported. |
|  | Derogatis Affects Balance Scale; DABS [[4](#_ENREF_4)]. | 40-item measure of recent positive and negative mood states and balance between them. Item scores are summed for joy, contentment, vigor, affection, anxiety, depression, guilt and hostility. An affect balance index (ranging from -4 to +4) can be calculated.  **Response format:** Retrospective 4-point Likert scale  **Designed as an outcome measure (Y/N)?** Y | Ω *  See [[4](#_ENREF_4)]. Instrument modified and examined in a longitudinal validation study involving 51 in-patients (average age = 74.8; n=36 women) with dementia (25 with Alzheimer’s dementia; 26 with vascular or mixed dementia). DABS subscales (e.g. positive affect) found to have acceptable test-retest reliability (r=0.62 to 0.87). Instrument discriminated depressed vs. non-depressed people with dementia and showed evidence of construct validity. |
|  | Positive and Negative Affect Scale; PANAS [[5](#_ENREF_5)]. | 20-item self-report measure of positive and negative affect (10 items each). Separate scores derived for negative (NA) and positive affect (PA).  **Response format:** Current, retrospective or general experience of specific emotions rated on 5-point Likert scale.  **Designed as an outcome measure (Y/N)?** N | Ω  PANAS used to evaluate a choral programme involving 28 older people (mean age 72.9; n=23 women), some of whom had dementia. No change in levels of positive or negative affect was detected by the instrument in response to this intervention [6]. |
| **Going Beyond** | Herth Hope Index; HHI ([7](#_ENREF_7)). | 12-item, self-report measure of particularised and generalised dimensions of hope in the present. Scale has 3 conceptually derived sub-scales; temporality and future; positive readiness and expectancy, and; interconnectedness. The sum of ratings yields global hope score.  **Response format:** Current - rating of agreement with statements on 4-point Likert scale.  **Designed as an outcome measure (Y/N)?** Y | Ω ^  HHI showed sensitivity to change (significant increase in hope) in a controlled study of the effectiveness of a spiritually-based reminiscence group programme amongst 103 (mean age = 73.6; n=71 women) Taiwanese community-dwelling PWD [8]. |
|  | Life Orientation Test; LOT-R [[9](#_ENREF_9)]. | 10-item measure of generalised optimism. Three items measure optimism and three measure the relative presence/absence of pessimism.  **Response format:** Rating of agreement statements about current sense of optimism; 4-point Likert scale  **Designed as an outcome measure (Y/N)?** N | *  LOT-R demonstrated adequate levels of internal consistency (α=0.63) with evidence of discriminant validity amongst a sample of 36 community dwelling people (mean age = 74.3; n=22 women) living with early stage dementia in the USA [[10](#_ENREF_10)]. |
|  | Meaning in Life Questionnaire; MLQ [[11](#_ENREF_11)]. | 10-item self-report measure of the current presence and search for existential meaning in life.  **Response format:** Respondents rate agreement statements on 7-point scale of 1 (‘absolutely untrue’) to 7 (‘absolutely true’). The measure yields separate scores for presence of and search for meaning.  **Designed as an outcome measure (Y/N)?** N | *  MLQ shown to have validity and good internal consistency (α = 0.77 to 0.84) amongst a sample of 36 community dwelling people (mean age = 74.3; n=22 women) living with early stage dementia in the USA [[10](#_ENREF_10)]. |
|  | Positive Psychology Outcome Measure; PPOM-Hope [[12](#_ENREF_12)]. | 16-item self-report measure of hope (and resilience; 8-item sub-scale for measuring hope.) in living with dementia. A mix of positively and negatively framed items are presented.  **Response format:** Retrospective (past month); 7-point Likert scale  **Designed as an outcome measure (Y/N)?** Y | *  Amongst 225 community dwelling PWD (mean age = 77.1; n=96 women) the PPOM (Hope) showed good levels of internal consistency (α=0.77) and evidence of convergent validity in dementia, e.g. negative correlation with a measure of depression [[12](#_ENREF_12)]. |
|  | Spirituality Index of Well-Being; SIWB [[13](#_ENREF_13)]. | 12-item self-report instrument that measures the presence of spiritual aspects of well-being in the present-day and which has two sub-scales; self-efficacy and life-scheme.  **Response format:** Statements about current spirituality are rated on a 5-point Likert scale  **Designed as an outcome measure (Y/N)?** Y | Ω^  SIWB used as an outcome measure and showed sensitivity to change (detected treatment effects) in a controlled study of the effectiveness of a spiritually-based reminiscence group programme amongst 103 (mean age = 73.6; n=71 women) Taiwanese community-dwelling PWD [8]. Psychometric properties in this sample were not reported. |
|  | Systems of Belief Inventory; SBI-15 [[14](#_ENREF_14)]. | 15-item measure of beliefs, practices, and perceptions of social support with respect spirituality and religiousness.  **Response format:** Brief statements about spiritual beliefs rated on four-point scale from 0 (strong disagree) to 3 (strongly agree). Some items require retrospective judgements.  **Designed as an outcome measure (Y/N)?** Unclear | Used in an exploratory mixed methods study with 23 PWD (mean age = 79; n=18 women). Showed association with quality of life scores. Psychometric properties not investigated [[15](#_ENREF_15)]. |
|  | Thriving of Older People Assessment Scale; TOPAS [[16](#_ENREF_16)]. | 32-item self or proxy-rated measure of perceived current thriving in relation to living in a residential care setting.  **Response format:** Items are rated in terms of agreement with current statements relating to thriving on a scale of 1 =disagree to 6 = completely agree  **Designed as an outcome measure (Y/N)?** Y | TOPAS used in a cross-sectional study with 192 nursing home residents (mean age = 86.9; 79% female); 80% living with dementia. Thriving scale showed correlations with level of functioning and quality of life [[17](#_ENREF_17)]. |
| **Agency & Purpose** | Bangor Goal Setting Interview; BGSI [[18](#_ENREF_18)]. | Structured interview for eliciting and self-rating the attainment of personally salient goals, including satisfaction with abilities in relation to goals.  **Response format:** Goal attainment self-rated on scale of 0-10. Can involve retrospective judgements.  **Designed as an outcome measure (Y/N)?** Y | Ω^  BGSI used in a multi-centre RCT of cognitive rehabilitation for PWD in the UK (n=475; n=226 women. Mean age = 78.5. 209 randomised to intervention). BGSI self-ratings at 3 and 9 months follow up showed significant positive effects for cognitive rehabilitation over treatment as usual [[19](#_ENREF_19)].  BGSI shows potential as a meaningful and acceptable person-centred outcome measure with PWD, particularly with respect to cognitive rehabilitation interventions. [[20](#_ENREF_20)] [[21](#_ENREF_21)]. |
|  | COOP/ WONCA charts [[22](#_ENREF_22)] | Developed to measure self-perceived functional status in-the-present across health and psycho-social domains, including physical fitness, feelings, social activities, pain and overall quality of life.  **Response format:** Concurrent items relating to domains rated on five-point rating scale.  **Designed as an outcome measure (Y/N)?** Y | *  Instrument validated with 67 people (mean age = 84.5; n=49 women) with moderate-severe dementia in institutional care able to be interviewed. Good inter-rater reliability (kappa = 0.9) but test-retest reliability over one-week period varied across domains and was ‘poor’ for quality of life and social activities. Some evidence for convergent and discriminant validity; 40% of hypothesised associations supported. Authors note that further modifications needed in dementia [[23](#_ENREF_23)]. |
|  | Decision Making Involvement Scale; DMI [[24](#_ENREF_24)]. | 15-item measure of perceived involvement in daily decision-making in the present. Developed specifically for people with dementia and their caregivers.  **Response format:** Agreement with statements rated on 4-point Likert scale from 0 (‘not at all involved’) to 3 (‘very involved’). Can be used as proxy-measure.  **Designed as an outcome measure (Y/N)?** N | *  DMI psychometric properties examined in 217 community-dwelling dyads in the USA. PWD had mean age of 76 (50% female). High levels of internal consistency reported. Supportive evidence of unitary factor structure and evidence of convergent and divergent validity; scores on DMI significantly associated with well-being outcomes (greater quality of life and less depression) [[24](#_ENREF_24)]. |
|  | Engagement and Independence in Dementia Questionnaire; EID-Q [[25](#_ENREF_25)]. | 26-item self-report measure of social engagement and sense of independence (two 13-item sub-scales). Developed with and for people with dementia.  **Response format:** Retrospective ratings of items ‘over the past month’ on a 5-point rating scale.  **Designed as an outcome measure (Y/N)?** Y | *  EID-Q reported to have good internal consistency (α=0.90) as well as evidence of convergent validity (e.g. positive and significant correlation with quality of life scores) amongst 225 community dwelling people with dementia in the UK (mean age = 77.1; n=96 women) [[25](#_ENREF_25)]. |
|  | Positive Psychology Outcome Measure; PPOM- Resilience [[12](#_ENREF_12)]. | 16-item self-report measure of resilience (&hope) developed with people with dementia. 8-item sub-scale for measuring resilience developed from qualitative work and using adapted items from the Connor-Davidson Resilience Scale.  **Response format:** Items are rated retrospectively (‘over the past month’? and according to 5-point rating scale.  **Designed as an outcome measure (Y/N)?** Y | *  Amongst 225 community dwelling PWD in the UK (mean age = 77.1; n=96 women), the PPOM (Resilience) showed good levels of internal consistency (α=0.84) and some evidence of convergent validity in dementia [[12](#_ENREF_12)]. |
|  | Resilience Scale; RS-14 [[26](#_ENREF_26), [27](#_ENREF_27)]. | 14-item self-report measure of individual resilience, defined in terms of adaptability and flexibility in the face of adversity. Items relate to current and general responses to adversity and cover optimism, agency, meaning and perseverance.  **Response format:** Items rated on 7-point scale of concurrent agreement (1 = disagree; 7 = agree).  **Designed as an outcome measure (Y/N)?** N | *  Psychometric properties of RS-14 examined amongst a sample of 36 people (mean age = 74.3; n=22 women) living with early stage dementia in the community in the USA. RS found to have good internal consistency (α=0.81) and adequate validity.[[10](#_ENREF_10)]. |
| **Positive Sense of Self** | General Self-Efficacy Scale; GSE [[28](#_ENREF_28)]. | 10-item self-report measure of general self-efficacy beliefs in the present.  Items rated on a Likert-type scale from ‘not at all true’ (1) to ‘exactly true’ (4).  **Designed as an outcome measure (Y/N)?** Y | Ω^  GSE sensitive to change (increased) in pilot RCT of a group intervention aimed at enhancing self-management abilities in 23 people (mean age = 75-76; n=6 women) with early stage dementia in the UK. Psychometric properties of GSE in this sample not reported [[29](#_ENREF_29)] |
|  | The Patient Dignity Inventory; PDI [[30](#_ENREF_30)]. | 25-item measure of aspects of dignity experience, e.g. appearance, emotions and relationships.  **Response format:** Items are concurrent statements about problems (i.e. lowered dignity) and rated on 5-point scale of subjective agreement. Higher scores indicate more perceived problems / less dignity.  **Designed as an outcome measure (Y/N)?** Y | Ω  PDI used in a mixed methods study of feasibility and effectiveness of dignity therapy in dementia. Study involved 7 people with early-stage dementia living in the community in the UK (mean age = 78.4; n=2 women). Improvements in self-reported dignity on the PDI noted for 2 / 7 participants [[31](#_ENREF_31)]. |
|  | The Rosenberg Self-Esteem Scale; RSES [[32](#_ENREF_32)]. | 10-item measure of current, positive and negative feelings / beliefs about oneself.  **Response format:** Items rated concurrently on 5-point Likert scale (1= ‘strongly agree’ to 5 ‘strongly disagree’).  **Designed as an outcome measure (Y/N)?** N | Ω  RSES used in a multi-modal intervention (combining cognitive-behavioural therapy with Taiji exercises and a support group) amongst 43 PWD in the USA (mean age = 76.9; n=20 women). RSES measured increased self-esteem in treatment group relative to control group following the intervention. Good internal consistency for the RSES reported for this sample (α=0.89-0.92) [[33](#_ENREF_33)].  RSES also used to evaluate a reminiscence intervention amongst 42 PWD in 2 nursing homes in Spain (mean age = 80.2; 69% female) but changes in self-esteem in the study not significant [[34](#_ENREF_34)]. |
|  | The Sherer Self-Efficacy Scale; SSES [[35](#_ENREF_35)]. | 23-item scale that measures current generalised and social self-efficacy perceptions.  **Response format:** Items are self-rated on Likert-type scales ranging from ‘strongly disagree’ to ‘strongly agree’.  **Designed as an outcome measure (Y/N)?** N | Ω  SSES used to evaluate a choral programme for a small sample of older people, some living with dementia [[6](#_ENREF_6)]. No changes in self-efficacy in response to this intervention found/detected. Psychometric properties of the SSES in this sample were not examined. |
|  | The Self-Identity in Dementia Questionnaire; SID-Q [[36](#_ENREF_36)]. | A structured assessment of identity roles and their current perceived value, in relation to 4 domains; occupational roles, family roles, leisure activities and attributes (23 question areas). Separate self-report and proxy versions are available.  **Response format:** Mix of Likert ratings (e.g. importance of specific roles / attributes rated 1-5) and yes/no responses (e.g. for specific activities)  **Designed as an outcome measure (Y/N)?** N | *  Authors of the SID-Q [[36](#_ENREF_36)] report some evidence of content/construct validity in a cross-sectional study of self-identity involving 38 PWD (mean age = 79.4; 76.3% female) in 2 nursing homes in Israel. Instrument used to measure role identity as a dependent variable amongst 50 PWD (+50 age-matched healthy controls) recruited from memory clinics in North Wales, UK (mean age = 77.8; n=26 women.) [[37](#_ENREF_37)]. Role identity strength was *partially* predicted by cognitive ability in relation to naming, constructional ability, word-list recognition, and autobiographical memory recall.  NB. [Cohen-Mansfield and colleagues [38](#_ENREF_38)] report good internal consistency and test-retest reliability for a *proxy-rated* version of the SID-Q. Participants in this study were 93 PWD – 41 attending day centres plus 53 residents from 2 nursing homes in Washington USA (71% female. Mean age = 87). |
|  | The Tennessee Self-Concept Scale; TSCS [[39](#_ENREF_39)]. | Originally, an 82-item self-report (or proxy) measure of self-concept. Contains subscales including positivity of identity (21-items) which can be extracted e.g. [[40](#_ENREF_40)]. Short 20-item version also developed [[39](#_ENREF_39)].  **Response format:** Items are concurrent statements (e.g. about roles) and rated on 5-point scale of subjective agreement.  **Designed as an outcome measure (Y/N)?** N | Ω  Used as an outcome variable in a longitudinal study of predictors of self-identity amongst 95 PWD presenting to memory clinics in the UK; mean age 78.5; n=49 women). Anxiety, depression and memory predicted TSCS scores over time [[41](#_ENREF_41)]. |
| **Connection and Belonging** | Brief Sense of Community Scale; BSCS [[42](#_ENREF_42)]. | 8-item measure of positive beliefs constituting a sense of community - includes perceived group membership, need fulfilment, influence, and emotional connections with others in the neighbourhood.  **Response format:** Items rated on a 5-point Likert-type scale ranging from ‘strongly agree’ to ‘strongly disagree’  **Designed as an outcome measure (Y/N)?** N | Ω  BSCS used in RCT of an intergenerational activity-based program with 40 Australian nursing home residents (plus 21 pre-school children), 50-65% of whom had a diagnosis of dementia (mean age = 91. 80% female). No treatment effects were detected by this scale in this study [[43](#_ENREF_43)]. |
|  | Gratitude Questionnaire; GQ-6 [[44](#_ENREF_44)]. | 6-item measure of experiences of social emotion of gratitude [gratefulness and appreciation] in everyday life.  **Response format:** Current agreement with gratitude statements; 7-point Likert scale  **Designed as an outcome measure (Y/N)?** N | *  GQ-6 found to have aspects of validity amongst a sample of 36 people (mean age = 74.3; n=22 women) living with early stage dementia in the community in the USA. Negatively correlated with measures of anxiety and depression but lower than expected internal consistency in this sample (α=0.56) as responses to the questionnaire appeared positively skewed [[10](#_ENREF_10)]. |
|  | Interpersonal Support  Evaluation List; ISEL 12 [[45](#_ENREF_45)]. | 12-item self-report measure of current, perceived availability of 3 aspects of social support; tangible, appraisal support, and belongingness. A 6-item version also available.  **Response format:** Items rated 1 (‘definitely false’) to 4 (definitely true). Higher summed scores indicate greater perceived availability of current social support.  **Designed as an outcome measure (Y/N)?** N | 6-item version of ISEL used as a *predictor* of hospitalisation in a prospective cohort study involving 2636 American older people. The study involved 471 PWD (mean age at inclusion = 75.5; 59% female) [[46](#_ENREF_46)]. |
|  | Lubben Social Network Scale; LSNS [[47](#_ENREF_47)]. | Self-report measure of social engagement and perceived social support. Standard (10-items), Short (6-items) and Expanded (18-items) versions can be utilised.  **Response format:** Items are rated in the here-and-now on Likert-type scales relating to frequency / extent of social contacts.  **Designed as an outcome measure (Y/N)?** Y | Ω  Japanese version of LSNS used as a secondary outcome measure in a controlled trial of a 12-week cooking rehabilitation intervention with 36 PWD in residential care (mean age = 85.4 +/- 6.5 years; n=29 women). No treatment effects detected; scores declined in intervention and control groups (i.e. smaller social network over time) [[48](#_ENREF_48)]. |
|  | Mutuality Scale; MS [[49](#_ENREF_49)]. | 15-item self-report measure of perceived quality of marital/close relationships, comprising affective closeness, shared values, shared pleasurable activities and reciprocity.  **Response format:** Items relate to perceived presence of domains in the person’s current experience of a relationship and rated from 1 (‘none’) to 4 (‘a great deal’).  **Designed as an outcome measure (Y/N)?** N | Ω  MS used in a longitudinal cohort study of 171 newly-diagnosed PWD living in the community in the USA (mean age = 76; 3% female). Higher levels of mutuality were associated with lower levels of depression and increased pleasant events over 4-month period [[50](#_ENREF_50)]. |
|  | Quality of Caregiver-Patient Relationships; QCPR [[51](#_ENREF_51)]. | 14-item self-report measure of general, perceived relationship quality within a caregiving relationship, from the perspective of either the carer or the care-recipient. High scores (>42) indicate the presence of affection, warmth and relationship satisfaction over perceived conflict and criticism.  **Response format:** Items rated concurrently on 5-point scale.  **Designed as an outcome measure (Y/N)?** Y | Ω  QCPR used to measure relationship quality as a dependent variable in a cohort study involving 50 people with dementia (and their carers). PWD had average age of 78.4; 40% female. Scores showed stability over 6-months but formal psychometric properties of the QCPR not explored in this sample [[52](#_ENREF_52)].  Used as a secondary outcome measure in RCT of a reminiscence intervention involving 488 community dwelling PWD in the UK (mean age 77; 47-52% female). No significant difference between treatment and control groups was evident [[53](#_ENREF_53)]. |
|  | Positive Affect Index; PAI [[54](#_ENREF_54)]. | 5-item measure of (current) positive affect held toward another person (e.g. care partner). Items cover communication quality, closeness, similarity of views on life, engagement in joint activities and overall perceived relationship quality. Response are rated on a 6-point scale from “not well” (1) to “extremely well” (6). Higher scores indicate higher relationship quality.  **Response format:** Items rated concurrently on 6-point scale.  **Designed as an outcome measure (Y/N)?** Unclear | *Ω  PAI used in a longitudinal study (18 months) of predictors of relationship quality (RQ) involving 54 dyads attending UK memory clinics, where one member had dementia (mean age 76.2; n=23 women). PAI treated as a dependent / criterion variable [[55](#_ENREF_55)].  PAI used to measure RQ towards care partners in a cross-sectional study involving 101 people with dementia (mean age 78.7; n=54 women) attending UK memory clinics [[56](#_ENREF_56)]. RQ partially predicted quality of life. The instrument has shown good internal consistency (Cronbach alpha 0.81) and re-test reliability over a 12-week period (r=0.66) amongst 77 PWD and their care partners (PWD; mean age = 77.5; n=44 women) [[5](#_ENREF_58)7]. |
| **Life Satisfaction – Valuing Life (also includes instruments relating to multiple domains)** | Control, Autonomy, Self-realisation & Pleasure Scale; CASP-19 [[5](#_ENREF_59)8]. | A measure of subjective quality of life (QoL) in the present. QoL is conceptualised as the perceived satisfaction of key psycho-social needs around control, autonomy, self-realisation (personal fulfilment and growth) and pleasure.  **Response format:** Items (some are negatively worded and reverse scored) rated on a 4-point scale from 0 (‘never’) to 3 (‘often’).  **Designed as an outcome measure (Y/N)?** Y | *  Psychometric properties of CASP examined amongst 225 community dwelling PWD in the UK (mean age 77.1; n=96 women). Factor structure was confirmed and the scale showed stability of scores over 1-week period with good levels of internal consistency overall (α=0.85). Total scores correlated negatively with measure of depression but positively with general quality of life scores, indicating convergent validity in dementia [[](#_ENREF_61)59]. Used in a population-based cohort study that included (n=51) PWD but their data not reported separately [[60](#_ENREF_60)]. |
|  | Life Satisfaction Index; LSI [[6](#_ENREF_62)1]. | Originally, a 20-item measure of perceived achievement and contentment with life. Proposed aspects of life satisfaction include zest (energy), resolution and fortitude, congruence between desired and achieved goals, positive self-concept and mood.  **Response format:** Response options are ‘disagree’, ‘don’t know’, or ‘agree’ (scored 0, 1 or 2). Some items are negatively worded  **Designed as an outcome measure (Y/N)?** Unclear | Ω^  Adapted 18-item version [[6](#_ENREF_62)2] showed sensitivity to change (significant increase in life satisfaction) in a controlled study of the effectiveness of a spiritually-based reminiscence group programme amongst 103 (mean age 73.6; n=71 women) Taiwanese community-dwelling PWD [8]. |
|  | Satisfaction with Life Scale; SLWS [[6](#_ENREF_63)3]. | A self-report measure of the *life satisfaction* component of subjective well-being. Five items assess perceived and current life satisfaction.  **Response format:** Each is rated on a 7-point Likert scale from 1 (‘strongly disagree’) to 7 (‘strongly agree’).  **Designed as an outcome measure (Y/N)?** Y | *  Psychometric properties of SLWS examined amongst a sample of 36 community dwelling people (mean age 74.3; n=22 women) living with early stage dementia in the USA [10]. Reported to possess aspects of validity for people with early-stage dementia as well as having acceptable levels of internal consistency (α=0.72). |
|  | Scales of Psychological Well-Being; SPWB [[64](#_ENREF_64)]. | Comprises six 14-item scales for measuring dimensions of psychological well-being; autonomy, environmental mastery, personal growth, positive relations with others, purpose in life, and self-acceptance.  **Response format:** Items rated concurrently on a six-point Likert scale  **Designed as an outcome measure (Y/N)?** Y | Ω^  SPWB detected improvements in aspects of psychological well-being (with the exception of purpose in life) in response to a reminiscence intervention amongst 42 PWD in 2 nursing homes in Spain (mean age 80.2; 69% female) [[34](#_ENREF_34)].  *  Adequate internal consistency (α=0.73) found for the *Purpose in Life* sub-scale amongst 91 community dwelling PWD in the USA (mean age 75.2; n=70 women) [[65](#_ENREF_65)]. |
|  | Short Warwick-Edinburgh Mental Well-Being Scale; SWEMWBS; [[66](#_ENREF_66)]. | 7 positively-worded items that assess aspects of subjective and psychological well-being and positive functioning in the preceding two weeks. Items cover feeling optimistic, feeling useful, feeling relaxed, dealing with problems, thinking clearly, feeling close to others and, decisiveness.  **Response format:** Items (e.g. *I have been feeling optimistic about the future)* are rated on a 5-point scale from 1 (‘none of the time’) to 5 (‘All of the time’).  **Designed as an outcome measure (Y/N)?** Y | Ω  Instrument used to evaluate the impact of peer support groups for PWD (n=21; mean age = 83; n=13 women) living in UK extra-care housing settings but did not show sensitivity to change and psychometric properties in this sample not examined [[67](#_ENREF_67)].  Used to evaluate group-based cognitive stimulation therapy (CST) in a UK memory clinic setting (mean age = 78; n=60; n=25 women). No significant difference in pre- to post- treatment scores found using this scale [[68](#_ENREF_68)]. |
|  | WHO-5 Well-being Index [[69](#_ENREF_69)]. | Comprises 5 positively worded items to measure different aspects of subjective well-being (cheerfulness, feeling calm, feeling active, good sleep, having interests) over the previous 2 weeks. Validated for elderly populations ([70](#_ENREF_70)).  **Response format:** Retrospective ratings on 6-point scale  **Designed as an outcome measure (Y/N)?** Y | Ω^  Adapted version of WHO-5 used in a randomised controlled trial of a recovery intervention in early-stage dementia in the UK (n= 48; mean age 78.4-79; n=32 women). Significant difference in WHO-5 scores over time in favour of treatment group vs. control [[71](#_ENREF_71)]. |

**References**

1. Tappen RM, Barry C. Assessment of Affect in Advanced Alzheimer's Diseases The Dementia Mood Picture Test. Journal of Gerontological Nursing. 1995 Mar 1;21(3):44-6.

2. Kolanowski A, Litaker M, Buettner L, Moeller J, Costa PT, Jr. A randomized clinical trial of theory-based activities for the behavioral symptoms of dementia in nursing home residents. Journal of the American Geriatrics Society. 2011;59(6):1032-41. Epub 2011/06/09.

3. Williams C, Tappen R, Wiese L, Newman D, Corbett M, Pinos S, Curtis B, Murray B. Stress in persons with dementia: Benefits of a memory center day program. Archives of psychiatric nursing. 2016 Oct 1;30(5):531-8.

4. Benedict RHB, Goldstein MZ, Derogatis LR. Assessment of Mood States in Psychiatrically Disturbed Patients With Dementia. The American journal of geriatric psychiatry : official journal of the American Association for Geriatric Psychiatry. 1996;4(4):298-310. Epub 1996/10/01.

5. Watson D, Clark LA, Tellegen A. Development and validation of brief measures of positive and negative affect: The PANAS scales. Journal of Personality and Social Psychology. 1988;54(6):1063-70.

6. Clements-Cortes AA. Buddy's Glee Club: Singing for Life. Activities, Adaptation & Aging. 2013;37(4):273-90.

7. Herth K. Abbreviated instrument to measure hope: development and psychometric evaluation. Journal of advanced nursing. 1992;17(10):1251-9.

8. Wu LF, Koo M. Randomized controlled trial of a six-week spiritual reminiscence intervention on hope, life satisfaction, and spiritual well-being in elderly with mild and moderate dementia. International journal of geriatric psychiatry. 2016;31(2):120-7. Epub 2015/05/13.

9. Scheier MF, Carver CS, Bridges MW. Distinguishing optimism from neuroticism (and trait anxiety, self-mastery, and self-esteem): A reevaluation of the Life Orientation Test. Journal of Personality and Social Psychology. 1994;67(6):1063-78.

10. McGee JS, Zhao HC, Myers DR, Kim SM. Positive Psychological Assessment and Early-Stage Dementia. Clinical Gerontologist. 2017;40(4):307-19.

11. Steger MF, Oishi S, Kashdan TB. Meaning in life across the life span: Levels and correlates of meaning in life from emerging adulthood to older adulthood. The Journal of Positive Psychology. 2009;4(1):43-52.

12. Stoner CR, Orrell M, Spector A. The Positive Psychology Outcome Measure (PPOM) for people with dementia: Psychometric properties and factor structure. Archives of gerontology and geriatrics. 2018;76:182-7. Epub 2018/03/13.

13. Daaleman TP, Frey BB, Wallace D, Studenski SA. Spirituality Index of Well-Being Scale: development and testing of a new measure. Journal of Family Practice. 2002;51(11):952.

14. Holland JC, Kash KM, Passik S, Gronert MK, Sison A, Lederberg M, et al. A brief spiritual beliefs inventory for use in quality of life research in life-threatening illness. Psycho-Oncology. 1998;7(6):460-9.

15. Katsuno T. Personal Spirituality of Persons with Early-Stage Dementia: Is it related to perceived quality of life? . Dementia. 2003;2(3):315-35.

16. Bergland A, Kirkevold M, Sandman PO, Hofoss D, Vassbo T, Edvardsson D. Thriving in long-term care facilities: instrument development, correspondence between proxy and residents' self-ratings and internal consistency in the Norwegian version. Journal of advanced nursing. 2014;70(7):1672-81. Epub 2013/12/10.

17. Patomella AH, Sandman PO, Bergland A, Edvardsson D. Characteristics of residents who thrive in nursing home environments: a cross-sectional study. Journal of advanced nursing. 2016;72(9):2153-61. Epub 2016/05/05.

18. Clare L, Nelis S, Kudlicka A. Bangor Goal-Setting Interview Manual2016 12/12/2017. Available from: https://psychology.exeter.ac.uk/media/universityofexeter/schoolofpsychology/reach/documents/The_Bangor_Goal-Setting_Interview_Version_2_Manual_(BGSI_v.2)_Dec_2016.pdf

19. Clare L, Kudlicka A, Oyebode JR, Jones RW, Bayer A, Leroi I, et al. Individual goal-oriented cognitive rehabilitation to improve everyday functioning for people with early-stage dementia: A multicentre randomised controlled trial (the GREAT trial). International journal of geriatric psychiatry. 2019. Epub 2019/02/07.

20. Clare L, Evans S, Parkinson C, Woods R, Linden D. Goal-Setting in Cognitive Rehabilitation for People with Early-Stage Alzheimer's Disease. Clinical Gerontologist. 2011;34(3):220-36.

21. Watermeyer TJ, Hindle JV, Roberts J, Lawrence CL, Martyr A, Lloyd-Williams H, et al. Goal Setting for Cognitive Rehabilitation in Mild to Moderate Parkinson's Disease Dementia and Dementia with Lewy Bodies. Parkinson's disease. 2016;2016:8285041. Epub 2016/07/23.

22. Van Weel C, Konig-Zahn C, Touw-Otten NP, Van Duijn NP, Meyboom-de Jong B. Measuring functional health status with the COOP/WONCA charts: A manual. Northern Centre of Health Care Research. 1995.

23. Ettema TP, Hensen E, De Lange J, Droes RM, Mellenbergh GJ, Ribbe MW. Self report on quality of life in dementia with modified COOP/WONCA charts. Aging & mental health. 2007;11(6):734-42. Epub 2007/12/13.

24. Menne HL, Tucke SS, Whitlatch CJ, Feinberg LF. Decision-making involvement scale for individuals with dementia and family caregivers. American journal of Alzheimer's disease and other dementias. 2008;23(1):23-9. Epub 2008/02/16.

25. Stoner CR, Orrell M, Spector A. Psychometric Properties and Factor Analysis of the Engagement and Independence in Dementia Questionnaire (EID-Q). Dementia and geriatric cognitive disorders. 2018;46(3-4):119-27. Epub 2018/09/05.

26. Wagnild GM, Young HM. Development and psychometric evaluation of the Resilience Scale. Journal of Nursing Measurement. 1993;1(2):165-78.

27. Wagnild GM. The Resilience Scale user's guide for the US English version of the Resilience Scale and the 14-Item Resilience Scale (RS-14). In: Center. TR, editor. Worden, MT 2009.

28. Schwarzer R, Jerusalem M. Generalized Self-Efficacy Scale. Weinman J, Wright S, Johnston M, editors. Windsor, England: NFER-NELSON; 1995.

29. Quinn C, Toms G, Jones C, Brand A, Edwards RT, Sanders F, et al. A pilot randomized controlled trial of a self-management group intervention for people with early-stage dementia (The SMART study). International psychogeriatrics. 2016;28(5):787-800. Epub 2015/12/18.

30. Chochinov HM, Hassard T, McClement S, Hack T, Kristjanson LJ, Harlos M, et al. The patient dignity inventory: a novel way of measuring dignity-related distress in palliative care. Journal of pain and symptom management. 2008;36(6):559-71. Epub 2008/06/27.

31. Johnston B, Lawton S, McCaw C, Law E, Murray J, Gibb J, et al. Living well with dementia: enhancing dignity and quality of life, using a novel intervention, Dignity Therapy. Int J Older People Nurs. 2016;11(2):107-20. Epub 2015/12/30.

32. Rosenberg M. Society and the adolescent self-image. Princeton, NJ: Princeton University Press.; 1965.

33. Burgener SC, Yang Y, Gilbert R, Marsh-Yant S. The effects of a multimodal intervention on outcomes of persons with early-stage dementia. American journal of Alzheimer's disease and other dementias. 2008;23(4):382-94. Epub 2008/05/06.

34. Gonzalez J, Mayordomo T, Torres M, Sales A, Melendez JC. Reminiscence and dementia: a therapeutic intervention. International psychogeriatrics. 2015;27(10):1731-7. Epub 2015/03/15.

35. Sherer M, Maddux JE, Mercandante B, Prentice-Dunn S, Jacobs B, Rogers RW. The Self-Efficacy Scale: Construction and Validation. Psychological Reports. 1982;51(2):663-71.

36. Cohen-Mansfield J, Golander H, Arnheim G. Self-identity in older persons suffering from dementia: preliminary results. Social science & medicine. 2000;51(3):381-94.

37. Caddell LS, Clare L. How does identity relate to cognition and functional abilities in early-stage dementia? Neuropsychology, development, and cognition Section B, Aging, neuropsychology and cognition. 2013;20(1):1-21. Epub 2012/02/22.

38. Cohen-Mansfield J, Parpura-Gill A, Golander H. Utilization of Self-Identity Roles for Designing Interventions for Persons With Dementia. The Journals of Gerontology Series B: Psychological Sciences and Social Sciences. 2006;61(4):P202-P12.

39. Fitts WHW, W. L. Tennessee self-concept scale: TSCS-2. Western psychological services. 1996.

40. Caddell LS, Clare L. A profile of identity in early-stage dementia and a comparison with healthy older people. Aging & mental health. 2013;17(3):319-27. Epub 2012/11/23.

41. Clare L, Whitaker CJ, Nelis SM, Martyr A, Markova IS, Roth I, et al. Self-concept in early stage dementia: profile, course, correlates, predictors and implications for quality of life. International journal of geriatric psychiatry. 2013;28(5):494-503.

42. Peterson NA, Speer PW, McMillan DW. Validation of A brief sense of community scale: Confirmation of the principal theory of sense of community. Journal of Community Psychology. 2008;36(1):61-73.

43. Low L-F, Russell F, McDonald T, Kauffman A. Grandfriends, an Intergenerational Program for Nursing-Home Residents and Preschoolers: A Randomized Trial. Journal of Intergenerational Relationships. 2015;13(3):227-40.

44. McCullough ME, Emmons RA, Tsang J-A. The grateful disposition: A conceptual and empirical topography. Journal of Personality and Social Psychology. 2002;82(1):112-27.

45. Cohen S, Mermelstein R, Kamarck T, Hoberman HM. Measuring the functional components of social support. In: Sarason I, Sarason B, editors. Social Support: Theory, Research and Applications. Seattle: Martinus Nijhoff 1985. p. 73-94.

46. Ennis SK, Larson EB, Grothaus L, Helfrich CD, Balch S, Phelan EA. Association of living alone and hospitalization among community-dwelling elders with and without dementia. Journal of general internal medicine. 2014;29(11):1451-9. Epub 2014/06/05.

47. Lubben J. Assessing social networks among elderly populations. Family & Community Health: The Journal of Health Promotion & Maintenance. 1988;11:42-52.

48. Murai T, Yamaguchi H. Effects of a Cooking Program Based on Brain-activating Rehabilitation for Elderly Residents with Dementia in a Roken Facility: A Randomized Controlled Trial. Progress in Rehabilitation Medicine. 2017;2(0):n/a.

49. Archbold PG, Stewart BJ, Greenlick MR, Harvath T. Mutuality and preparedness as predictors of caregiver role strain. Research in Nursing & Health. 1990;13(6):375-84.

50. Ball V, Snow AL, Steele AB, Morgan RO, Davila JA, Wilson N, et al. Quality of relationships as a predictor of psychosocial functioning in patients with dementia. Journal of geriatric psychiatry and neurology. 2010;23(2):109-14. Epub 2010/03/18.

51. Spruytte N, Van Audenhove C, Lammertyn F, Storms G. The quality of the caregiving relationship in informal care for older adults with dementia and chronic psychiatric patients. Psychology and psychotherapy. 2002;75(Pt 3):295-311. Epub 2002/10/25.

52. Spector A, Orrell M, Charlesworth G, Marston L. Factors influencing the person-carer relationship in people with anxiety and dementia. Aging & mental health. 2016;20(10):1055-62. Epub 2015/07/25.

53. Woods RT, Orrell M, Bruce E, Edwards RT, Hoare Z, Hounsome B, et al. REMCARE: Pragmatic Multi-Centre Randomised Trial of Reminiscence Groups for People with Dementia and their Family Carers: Effectiveness and Economic Analysis. PloS one. 2016;11(4):e0152843. Epub 2016/04/20.

54. Bengtson VL. Positive Affect Index: Subjective solidarity between parents and children. Research instruments in social gerontology. 1982:129-33.

55. Clare L, Nelis SM, Whitaker CJ, Martyr A, Markova IS, Roth I, et al. Marital relationship quality in early-stage dementia: perspectives from people with dementia and their spouses. Alzheimer disease and associated disorders. 2012;26(2):148-58. Epub 2011/06/21.

56. Woods RT, Nelis SM, Martyr A, Roberts J, Whitaker CJ, Markova I, et al. What contributes to a good quality of life in early dementia? Awareness and the QoL-AD: a cross-sectional study. Health and quality of life outcomes. 2014;12:94. Epub 2014/06/13.

57. Woods RT. Relationship quality and quality of life in dementia. 19th International Congress of Gerontology; Paris2009.

58. Hyde M, Wiggins RD, Higgs P, Blane DB. A measure of quality of life in early old age: the theory, development and properties of a needs satisfaction model (CASP-19). Aging & mental health. 2003;7(3):186-94. Epub 2003/05/31.

59. Llewellyn DJ, Lang IA, Langa KM, Huppert FA. Cognitive function and psychological well-being: findings from a population-based cohort. Age and ageing. 2008;37(6):685-9. Epub 2008/10/15.

60. Stoner CR, Orrell M, Spector A. The psychometric properties of the control, autonomy, self-realisation and pleasure scale (CASP-19) for older adults with dementia. Aging & mental health. 2018:1-7. Epub 2018/01/23.

61. Neugarten BL, Havighurst RJ, Tobin SS. The Measurement of Life Satisfaction. Journal of Gerontology. 1961;16(2):134-43.

62. Diener E, Emmons RA, Larsen RJ, Griffin S. The Satisfaction With Life Scale. Journal of personality assessment. 1985;49(1):71-5. Epub 1985/02/01.

63. Ryff CD, Keyes CLM. The structure of psychological well-being revisited. Journal of Personality and Social Psychology. 1995;69(4):719-27.

64. Mak W. Self-reported goal pursuit and purpose in life among people with dementia. The journals of gerontology Series B, Psychological sciences and social sciences. 2010;66(2):177-84. Epub 2010/12/29.

65. Stewart-Brown S, Tennant A, Tennant R, Platt S, Parkinson J, Weich S. Internal construct validity of the Warwick-Edinburgh Mental Well-being Scale (WEMWBS): a Rasch analysis using data from the Scottish Health Education Population Survey. Health and quality of life outcomes. 2009;7:15. Epub 2009/02/21.

66. Chakkalackal L. The value of peer support on cognitive improvement amongst older people living with dementia. Research. Policy and Planning. 2015;31(2):127-41.

67. Allward C, Dunn R, Forshaw G, Rewston C, Wass N. Mental wellbeing in people with dementia following Cognitive Stimulation Therapy: Innovative practice. Dementia. 2017:1471301217722443. Epub 2017/07/28.

68. World Health Organization(WHO). Wellbeing Measures in Primary Health Care: The DepCare Project. WHO, Regional Office for Europe, Copenhagen: 1998.

69. Heun R, Burkart M, Maier W, Bech P. Internal and external validity of the WHO Well-Being Scale in the elderly general population. Acta psychiatrica Scandinavica. 1999;99(3):171-8. Epub 1999/04/01.

70. Jha A, Jan F, Gale T, Newman C. Effectiveness of a recovery-orientated psychiatric intervention package on the wellbeing of people with early dementia: a preliminary randomised controlled trial. International journal of geriatric psychiatry. 2013;28(6):589-96. Epub 2012/08/01.

1. PPOM sub-scales treated as separate measures as psychometric properties have been established independently for each (see Stoner, 2018a). [↑](#footnote-ref-1)
